# Development and validation of a uric acid-inflammation-metabolism score for predicting osteoarthritis risk: evidence from NHANES 2007–2018 and an external Chinese cohort

**DOI:** 10.3389/fendo.2026.1861185

**Published:** 2026-06-18

**Authors:** Chengyin Lu, Zhe Yang, Yongqin Ji, Zhuoyue Song, Liwei Wei, Xiaohui Wang

**Affiliations:** 1 Department of Sports Medicine II, Henan Orthopaedic Hospital, Luoyang Orthopaedic Hospital, Zhengzhou, Henan, China; 2 Department of Emergency, The First Affiliated Hospital of USTC, Division of Life Sciences and Medicine, University of Science and Technology of China, Hefei, Anhui, China

**Keywords:** composite score, inflammation, LASSO regression, metabolic disturbance, osteoarthritis, risk prediction, uric acid

## Abstract

**Objective:**

To develop and validate a novel Uric acid-Inflammation-Metabolism (UIM) score for assessing the association with osteoarthritis(OA) prevalence and to explore joint effect mechanisms among three biological dimensions: uric acid metabolism, inflammatory status, and metabolic disturbance.

**Methods:**

This retrospective cross-sectional study included a discovery cohort from NHANES 2007–2018 (n=3288) and an external validation cohort from Anhui Provincial Hospital (n=859). LASSO logistic regression selected optimal predictors from 14 candidate variables, and the UIM score was constructed via weighted multivariable logistic regression. Discrimination, calibration, and clinical utility were evaluated, with restricted cubic splines (RCS) assessing dose-response relationships and additive interactions analyzed.

**Results:**

Seven variables (UA, eGFR, serum creatinine, hs-CRP, WC, HDL-C, BMI) were retained. In the fully adjusted model, participants in the highest UIM score quartile (Q4) had a 2.63-fold higher OA risk than those in the lowest quartile (Q1) (OR = 2.63, 95% CI: 1.47–4.72), with a significant J-shaped nonlinear relationship. The UIM score (AUC = 0.707) outperformed individual biomarkers and the traditional model, with satisfactory calibration and clinical utility. The uric acid dimension contributed most (70.50%), followed by metabolic (26.92%) and inflammatory (2.58%) dimensions. A significant additive antagonistic interaction was observed between high inflammation and high metabolic disturbance. External validation and sensitivity analyses confirmed the score’s robustness and generalizability.

**Conclusions:**

The UIM score integrating uric acid metabolism, inflammation, and metabolic disturbance outperforms individual biomarkers in OA risk prediction with good cross-cohort generalizability. Antagonistic interactions among the three dimensions reflect the complexity of OA pathogenesis, and the UIM score is a practical tool for OA prevalence stratification and clinical assessment.

## 1 Introduction

Osteoarthritis (OA) is the most prevalent degenerative joint disease globally, affecting over 528 million people worldwide. Its prevalence continues to rise with population aging and the obesity epidemic (1). OA is characterized by progressive cartilage degeneration, subchondral bone remodeling, and synovial inflammation. It causes joint pain, functional impairment, and reduced quality of life, and is one of the leading causes of disability in the elderly (2). Data from the Global Burden of Disease Study 2019 showed that OA-related disability-adjusted life years increased by 48% compared to 1990. This imposes a substantial economic burden on healthcare systems worldwide (3). However, tools for identifying and stratifying OA prevalence remain limited. In clinical practice, diagnosis is often made only after irreversible joint structural damage has occurred, missing the optimal intervention window (4). Developing convenient, cost-effective, and reliable OA risk assessment tools is therefore of great clinical and public health significance.

In recent years, the concept of “metabolic osteoarthritis” has attracted widespread attention (5). Growing evidence indicates that OA is not merely a localized mechanical wear-and-tear disease, but a systemic condition closely linked to whole-body metabolic dysfunction (6). Among various metabolic factors, abnormal uric acid metabolism, chronic low-grade inflammation, and metabolic syndrome are recognized as three key dimensions in OA pathogenesis (7–9).

The relationship between serum uric acid (SUA) and OA has long been controversial. On one hand, hyperuricemia can cause monosodium urate crystal deposition in articular cartilage and synovial tissues. This activates the NLRP3 inflammasome pathway and promotes the release of pro-inflammatory mediators such as interleukin-1β (IL-1β), thereby accelerating joint inflammation and cartilage degeneration (10). On the other hand, uric acid has antioxidant properties at physiological concentrations, scavenging approximately 60% of plasma free radicals. Theoretically, this provides protective effects on articular cartilage (11).

This “double-edged sword” effect suggests that uric acid’s impact on OA may involve nonlinear relationships. Thus, risk assessment based solely on uric acid levels may be insufficient. Furthermore, uric acid metabolism is closely associated with renal function. Decreased glomerular filtration rate can reduce uric acid excretion, further worsening hyperuricemia (12). Integrating uric acid levels with renal function indices may therefore more comprehensively reflect uric acid metabolic status.

Chronic low-grade inflammation is a core pathological mechanism in OA development. High-sensitivity C-reactive protein (hs-CRP), the most widely used systemic inflammatory biomarker, has been consistently linked to OA prevalence risk and disease progression in multiple epidemiological studies (13). Elevated hs-CRP reflects systemic inflammatory activation, which promotes matrix metalloproteinase (MMP) expression, inhibits chondrocyte anabolic metabolism, and accelerates articular cartilage degradation (14).

Additionally, decreased serum albumin levels are not only an indicator of malnutrition but also an important marker of chronic inflammatory consumption. Hypoalbuminemia is closely associated with sarcopenia, bone loss, and periarticular supportive tissue degeneration. It may indirectly promote OA development by reducing joint stability (15). However, the predictive value of individual inflammatory markers is limited, and integrating multiple inflammatory indices may provide a more comprehensive assessment of inflammatory status.

Metabolic syndrome (MetS) and its components including hyperglycemia, dyslipidemia, and central obesity have been identified as independent risk factors for OA (16). Insulin resistance can lead to the accumulation of advanced glycation end-products (AGEs) in articular cartilage, inducing chondrocyte oxidative stress and apoptosis (17). Hypertriglyceridemia promotes lipid deposition in joint tissues, activating lipid-mediated inflammatory pathways (18).

Obesity affects OA through both mechanical loading and metabolic mechanisms. Excessive weight increases joint loading pressure, while adipokines secreted by adipose tissue (such as leptin and adiponectin) directly participate in joint inflammation and cartilage metabolic regulation (19). The triglyceride-glucose (TyG) index, a surrogate marker for insulin resistance, has received widespread attention, though its predictive capacity for OA remains to be improved.

Notably, complex interactions exist among these three dimensions. Hyperuricemia can exacerbate insulin resistance (20), while insulin resistance can further increase serum uric acid by reducing renal uric acid excretion (21). Inflammation acts as both a consequence and an amplifier of metabolic dysfunction (22). This three-dimensional positive feedback loop suggests that evaluating individual dimensions in isolation may underestimate actual OA risk. Integrating all three dimensions into a comprehensive score may more accurately capture the pathophysiological characteristics of metabolic OA.

However, no studies have systematically integrated uric acid, inflammatory, and metabolic dimensions to construct OA risk prediction scores. Existing research has mainly focused on single biomarkers or single-dimension analyses (23, 24), most of which lack external validation. This leaves the association performance and generalizability of these analyses inadequately assessed. Moreover, metabolic characteristics vary across different racial and geographic populations, and the cross-ethnic applicability of OA risk prediction models remains unclear.

Based on this background, the present study proposes the following scientific hypotheses (1): a comprehensive UIM score, integrating uric acid metabolism, inflammatory status, and metabolic disturbance, is independently associated with OA prevalence risk (2); the UIM score’s OA predictive performance is superior to that of any single biomarker (3); this association is consistent across different racial populations. To test these hypotheses, this study used the NHANES database (a multi-ethnic U.S. population) to construct the UIM score via LASSO regression and multivariable logistic regression, followed by external validation. The aim is to provide a novel tool for early screening and risk stratification of metabolic OA.

## 2 Methods

### 2.1 Study design and data sources

This retrospective cross-sectional study utilized data from two independent sources: the National Health and Nutrition Examination Survey (NHANES) and Anhui Provincial Hospital. NHANES is a nationally representative cross-sectional survey conducted by the National Center for Health Statistics (NCHS) of the Centers for Disease Control and Prevention (CDC). It employs a complex multistage probability sampling design to continuously survey the non-institutionalized civilian US population, collecting comprehensive information on demographic characteristics, health status, laboratory examinations, and physical measurements. The present study incorporated data from six survey cycles spanning 2007–2008, 2009–2010, 2011–2012, 2013–2014, 2015–2016, and 2017–2018. All NHANES data were approved by the NCHS Ethics Review Committee, with informed consent obtained from all participants. Data are publicly accessible through the official NHANES website (https://www.cdc.gov/nchs/nhanes/). This study used public NHANES data for primary analysis, and no additional ethical approval was required. The external validation was a retrospective study approved by the institutional ethics committee, and informed consent was waived due to retrospective design and anonymous data processing. All procedures complied with the Declaration of Helsinki.

### 2.2 Study population

#### 2.2.1 NHANES discovery cohort

**Figure 1:**
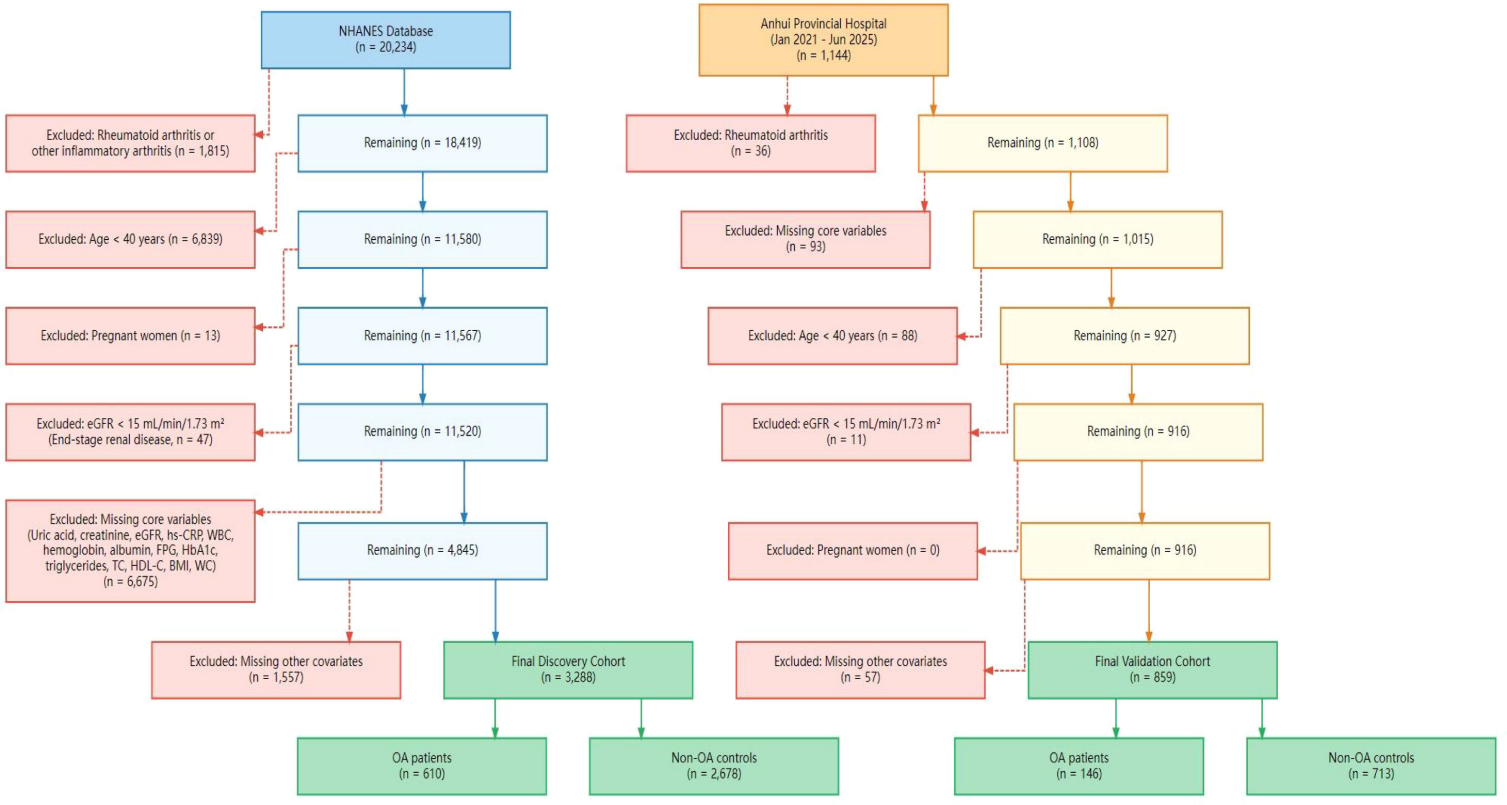
Study flow chart.

Inclusion criteria (1): Age ≥40 years (2); completion of Mobile Examination Center (MEC) physical examination and laboratory testing (3); complete osteoarthritis diagnostic information. Exclusion criteria (1): Self-reported history of rheumatoid arthritis or other inflammatory arthritides (n = 1,815) (2); age <40 years (n = 6,839) (3); pregnant women (n = 13) (4); estimated glomerular filtration rate (eGFR) <15 mL/min/1.73 m² (end-stage renal disease, n = 47), due to severely altered uric acid metabolism (5); missing core variables (serum uric acid, serum creatinine, eGFR, hs-CRP, white blood cell count, hemoglobin, serum albumin, fasting plasma glucose, glycated hemoglobin, triglycerides, total cholesterol, high-density lipoprotein cholesterol, BMI, and waist circumference) (n = 6,675) (6); missing other covariates (n = 1,557).After screening, 3,288 participants were ultimately included as the discovery cohort, comprising 610 OA patients and 2,678 non-OA controls. The study population selection flowchart is presented in **Figure 1**.

#### 2.2.2 Validation cohort

Inpatients admitted to Anhui Provincial Hospital between January 2021 and June 2025 were selected as study subjects. Inclusion criteria (1): Age ≥40 years (2); completion of blood biomarker testing (3); complete osteoarthritis diagnostic information. Exclusion criteria (1): Rheumatoid arthritis (n = 36) (2); missing core variable data (n = 93) (3); age <40 years (n = 88) (4); eGFR <15 mL/min/1.73 m² (n = 11) (5); pregnant women (n = 0) (6); missing other covariates (n = 57).Finally, 859 participants were included as the validation cohort, comprising 146 osteoarthritis patients and 713 non-osteoarthritis controls. The study population selection flowchart is detailed in **Figure 1**.

### 2.3 Variable definitions

#### 2.3.1 Outcome variables

OA definition in NHANES: Based on questionnaire MCQ160a (“Has a doctor or other health professional ever told you that you had arthritis?”), participants responding “Yes” were further classified according to MCQ195 (“Which type of arthritis?”). Those selecting “Osteoarthritis” or “Degenerative arthritis” were defined as having OA. Participants responding “No” or having non-OA types were classified as non-OA controls. OA diagnosis in the validation cohort: Diagnosis followed the American College of Rheumatology (ACR) criteria (25).OA diagnostic criteria (1):Pain in the past month (2);radiographic evidence of marginal osteophytes (3); synovial fluid consistent with OA characteristics (clear, viscous, white blood cell count <2,000/μL) (4); age ≥40 years (5); morning stiffness <30 minutes (6); crepitus on motion. Diagnosis required meeting (1)+ (2), or (1)+ (3)+ (5)+ (6), or (1)+ (4)+ (5)+ (6). Hip and hand OA diagnoses similarly followed ACR criteria. All patients were diagnosed by rheumatology or orthopedic specialists, with radiographic confirmation of affected joints.

#### 2.3.2 Candidate predictor variables

This study selected 14 candidate variables across three dimensions—uric acid metabolism, inflammatory status, and metabolic disturbance—all cross-validated for availability in both databases. Uric acid dimension (3 variables) (1): Serum uric acid (SUA), NHANES variable LBXSUA, unit: mg/dL (2); serum creatinine, NHANES variable LBXSCR, unit: mg/dL (3); estimated glomerular filtration rate (eGFR), calculated using the CKD-EPI equation based on creatinine, age, and sex, unit: mL/min/1.73 m².Inflammatory dimension (4 variables) (1): High-sensitivity C-reactive protein (hs-CRP), NHANES variable LBXHSCRP, unit: mg/L (2); white blood cell count (WBC), NHANES variable LBXWBCSI, unit: 10³/μL (3); hemoglobin (HGB), NHANES variable LBXHGB, unit: g/dL (4); serum albumin, NHANES variable LBXSAL, unit: g/dL. Metabolic dimension (5 variables) (1): Fasting plasma glucose (FPG), NHANES variable LBXGLU, unit: mg/dL (2); glycated hemoglobin (HbA1c), NHANES variable LBXGH, unit: % (3); triglycerides (TG), NHANES variable LBXSTR, unit: mg/dL (4); total cholesterol (TC), NHANES variable LBXTC, unit: mg/dL (5); high-density lipoprotein cholesterol (HDL-C), NHANES variable LBDHDD, unit: mg/dL. Additionally, body mass index (BMI) and waist circumference (WC) were included as obesity indicators within the metabolic dimension. BMI was calculated from height and weight (kg/m²), and WC was recorded in centimeters.

#### 2.3.3 Covariates

Based on previous literature and clinical experience, the following variables were selected as potential confounders for adjustment: Demographic variables: Age (continuous variable, years), sex (male/female), race/ethnicity (NHANES: non-Hispanic White, non-Hispanic Black, Mexican American, other races; not applicable for validation cohort), education level (≤middle school, high school, ≥college), marital status (married/cohabiting, other).Lifestyle variables: Smoking status (never smoker, former smoker, current smoker), alcohol consumption status (never drinker, former drinker, current drinker), physical activity level (low, moderate, high, classified according to WHO standards).Comorbidities: Diabetes mellitus (NHANES: self-reported physician diagnosis, or FPG ≥126 mg/dL, or HbA1c ≥6.5%; validation cohort: self-reported physician diagnosis or FPG ≥126 mg/dL), hypertension (NHANES: self-reported physician diagnosis, or systolic blood pressure ≥140 mmHg, or diastolic blood pressure ≥90 mmHg).

### 2.4 Statistical analysis

For variables with missing proportions <5%, multiple imputation using chained equations (MICE) was applied (m = 5 imputations).Continuous variables with skewed distributions underwent natural logarithmic transformation to improve normality. All continuous variables included in LASSO regression and score construction were standardized using Z-scores. Continuous variables are presented as mean (SE), while categorical variables are expressed as % (SE). Baseline characteristics between groups were compared using one-way ANOVA for continuous variables and Pearson chi-square tests for categorical variables. All NHANES analyses incorporated complex sampling design elements, including weights (MEC weights), strata, and primary sampling units (PSU) to obtain nationally representative estimates. Analysis weights across multiple survey cycles were combined and adjusted according to NHANES analytical guidelines.

UIM Score Construction: LASSO (Least Absolute Shrinkage and Selection Operator) logistic regression was employed to select the optimal variable combination from 14 candidates for the UIM score. LASSO introduces an L1 penalty term (λ∑|βj|) into maximum likelihood estimation, shrinking coefficients of unimportant variables to zero, thereby achieving variable selection and model parsimony. Ten-fold cross-validation was used to select the optimal penalty parameter λ, preferentially choosing lambda.1se (the largest λ such that the cross-validation error is within one standard error of the minimum) to obtain a more parsimonious model. Variables with non-zero coefficients were retained for subsequent UIM score construction. Based on LASSO-selected variables, a weighted multivariable logistic regression model was fitted. The UIM score calculation formula was: UIM Score = β_1_ ×Z_1_ + β_2_ ×Z_2_ +… + β_k_ ×Z_k_, where β_i_ represents the regression coefficient of the ith variable in logistic regression, Z_i_ represents the corresponding variable’s Z-score standardized value, and k represents the number of variables ultimately included.

Association Analysis: Weighted multivariable logistic regression was used to analyze the association between UIM score and OA prevalence risk, constructing four progressively adjusted models: Model 1 (unadjusted): UIM score only; Model 2: adjusted for age and sex; Model 3: Model 2 plus education level, smoking status, alcohol consumption, and physical activity level; Model 4 (fully adjusted): Model 3 plus diabetes mellitus, hypertension, and CVD history. Results are presented as odds ratios (OR) with 95% confidence intervals (95% CI). The UIM score was analyzed both as a continuous variable (per 1-SD increase) and as a categorical variable (Q1–Q4 quartiles). Linear trends across quartiles were tested using P for trend by treating quartile groups as continuous variables in the model. Dose-Response Analysis: Restricted cubic splines (RCS) were used to analyze the dose-response relationship between UIM score and OA risk. Spline knots were set at 3–5 locations (optimal number selected based on Akaike Information Criterion), with the reference point set at the median UIM score. P-overall (overall association) and P-nonlinear (nonlinear relationship) were tested separately, with P-nonlinear <0.05 indicating significant nonlinearity.

Subgroup Analysis: Prespecified subgroup analyses were conducted by: age (<60 vs ≥60 years), sex (male vs female), BMI (<25 vs 25–30 vs ≥30 kg/m²), diabetes mellitus (yes vs no), hypertension (yes vs no), smoking status (never vs former/current), and eGFR (≥60 vs <60 mL/min/1.73 m²). Interaction effects were tested by including interaction terms between subgroup variables and UIM score in logistic regression models, with interaction P <0.05 considered statistically significant. Results are presented as forest plots.

Predictive Performance Assessment: Receiver operating characteristic (ROC) curves were plotted and area under the curve (AUC) calculated. AUCs of the UIM score and individual indicators (uric acid, eGFR, hs-CRP, BMI, WC, creatinine, and HDL) were compared using DeLong tests to assess statistical significance of differences. Additionally, net reclassification improvement (NRI) and integrated discrimination improvement (IDI) were calculated to evaluate the incremental association value of the UIM score compared with traditional risk factor models. Model Calibration: Hosmer-Lemeshow goodness-of-fit test assessed consistency between predicted and observed probabilities (P >0.05 indicating good calibration). Calibration curves were plotted, and mean absolute error (MAE), calibration slope, and calibration intercept were calculated. Clinical Utility Assessment: Decision curve analysis (DCA) evaluated the clinical net benefit of the UIM score across different threshold probabilities, comparing it with individual indicator models and “treat all” versus “treat none” strategies.

Dimensional Contribution Analysis: Sub-scores for the three dimensions (uric acid, inflammation, and metabolism) were calculated: for each dimension, sub-score = β_1_ ×Z_1_ + β_2_ ×Z_2_ +… + β_n_ ×Z_n_ (all variables within that dimension). Association strength (OR) between each dimensional sub-score and OA was calculated separately, with relative contribution proportions visualized using stacked bar charts. Additionally, interdimensional interaction effects were explored, testing whether joint effects of high uric acid × high inflammation, high uric acid × high metabolic disturbance, and high inflammation × high metabolic disturbance exhibited super-additive interactions.

External Validation: Regression coefficients and standardization parameters (means and standard deviations) obtained from NHANES modeling were directly applied to the external validation dataset (1): variables in external validation were unified to units consistent with NHANES (2); external validation variables were Z-score standardized using NHANES means and standard deviations (3); UIM scores for each external validation participant were calculated using NHANES-derived coefficients. Primary association analyses (logistic regression), discrimination assessment (AUC), calibration assessment, and subgroup analyses were repeated in external validation to evaluate UIM score performance.

Sensitivity Analyses: To assess result robustness, the following sensitivity analyses were conducted (1): re-analysis after excluding participants with hs-CRP >10 mg/L (possible acute infection) (2); re-analysis after excluding participants with BMI <18.5 kg/m² (underweight) (3); re-analysis after substituting eGFR calculation formula (CKD-EPI 2021 creatinine equation) (4); re-analysis using inverse probability of treatment weighting (IPTW) to further balance confounding factors between groups (5); random split of NHANES discovery cohort into 70% training and 30% internal validation sets to assess model internal stability. All statistical analyses were performed using R software (version 4.3.2). Two-sided P <0.05 was considered statistically significant.

## 3 Results

### 3.1 Baseline characteristics of the study population

The NHANES discovery cohort included 3,288 participants, comprising 610 individuals with OA and 2,678 non-OA controls. **Table 1** presents the baseline characteristics comparison between the two groups. The OA group was significantly older than the non-OA group [62.31(0.50) vs. 56.25(0.40) years, P < 0.0001], had a higher proportion of females [63.31(2.96) vs. 44.08(1.44), P < 0.0001], a higher percentage of non-Hispanic whites [82.41(1.64) vs. 69.87(1.79), P < 0.0001], a greater proportion of “other” marital status (including widowed) [19.83(2.33) vs. 12.45(1.02), P = 0.01], and significant differences in educational attainment distribution (P = 0.03).Regarding renal function parameters, the OA group had significantly lower eGFR compared to the non-OA group [81.95(0.93) vs. 87.97(0.67) mL/min/1.73 m², P < 0.0001], as well as lower serum creatinine [76.98(1.16) vs. 80.31(0.57) μmol/L, P = 0.04] and uric acid levels [317.93(4.91) vs. 327.69(2.55) μmol/L, P = 0.03]. In terms of metabolic parameters, the OA group exhibited significantly higher BMI [30.21(0.38) vs. 29.19(0.24) kg/m², P = 0.02] and waist circumference [103.55(0.92) vs. 101.41(0.63) cm, P = 0.04].For lipid and hematological parameters, the OA group demonstrated higher total cholesterol [5.17(0.07) vs. 4.98(0.04) mmol/L, P = 0.02] and HDL-C [1.54(0.04) vs. 1.42(0.02) mmol/L, P = 0.002], while hemoglobin levels were lower [14.27(0.07) vs. 14.46(0.05) g/dL, P = 0.01]. Regarding the composite biomarker, the OA group had significantly higher UIM scores compared to the non-OA group [-1.15(0.03) vs. -1.65(0.03), P < 0.0001], with a significantly higher proportion in the Q4 quartile [45.51(2.63) vs. 24.75(1.49), P < 0.0001]. Additionally, the OA group had significantly higher prevalences of hypertension [59.22(2.90) vs. 43.79(1.75)] and cardiovascular disease [17.98(2.39) vs. 9.02(0.89)] (both P < 0.001). No statistically significant differences were observed between groups for HbA1c, fasting glucose, triglycerides, WBC count, hs-CRP, albumin, diabetes, smoking, alcohol consumption, or physical activity levels (all P > 0.05).

**Table 1:** Baseline characteristics of study population.

Variables	Total(n=3288)	Non-OA(n=2678)	OA(n=610)	P-value
Age,mean (SE)	57.53(0.37)	56.25(0.40)	62.31(0.50)	< 0.0001
Creatinine,mean (SE)	80.03(0.50)	80.31(0.57)	76.98(1.16)	0.04
UA,mean (SE)	325.63(2.32)	327.69(2.55)	317.93(4.91)	0.03
BMI,mean (SE)	29.41(0.22)	29.19(0.24)	30.21(0.38)	0.02
WC,mean (SE)	101.87(0.56)	101.41(0.63)	103.55(0.92)	0.04
eGFR,mean (SE)	86.70(0.60)	87.97(0.67)	81.95(0.93)	< 0.0001
HbA1c,mean (SE)	5.82(0.02)	5.81(0.03)	5.86(0.05)	0.36
WBC,mean (SE)	6.72(0.06)	6.67(0.07)	6.91(0.12)	0.12
FPG,mean (SE)	6.29(0.05)	6.28(0.06)	6.31(0.09)	0.78
HB,mean (SE)	14.42(0.05)	14.46(0.05)	14.27(0.07)	0.01
TG,mean (SE)	1.34(0.03)	1.33(0.03)	1.40(0.06)	0.29
TC,mean (SE)	5.02(0.04)	4.98(0.04)	5.17(0.07)	0.02
HDL,mean (SE)	1.45(0.01)	1.42(0.02)	1.54(0.04)	0.002
hs-CRP,mean (SE)	3.59(0.15)	3.48(0.17)	3.98(0.36)	0.23
Albumin,mean (SE)	4.13(0.01)	4.13(0.01)	4.10(0.02)	0.15
UIM,mean (SE)	-1.54(0.02)	-1.65(0.03)	-1.15(0.03)	< 0.0001
Sex,%(SE)				< 0.0001
Female	48.14(0.02)	44.08(1.44)	63.31(2.96)	
Male	51.86(0.03)	55.92(1.44)	36.69(2.96)	
Race,%(SE)				< 0.0001
Mexican American	6.27(0.01)	7.14(0.87)	2.99(0.53)	
Non-Hispanic Black	8.01(0.01)	8.55(0.91)	5.98(0.95)	
Non-Hispanic White	72.52(0.04)	69.87(1.79)	82.41(1.64)	
Other	13.21(0.01)	14.43(1.08)	8.62(1.36)	
Marital,%(SE)				0.01
Divorced	9.69(0.01)	9.83(0.85)	9.17(2.02)	
Married	69.46(0.04)	70.35(1.64)	66.13(3.36)	
Never married	6.84(0.01)	7.37(0.90)	4.87(1.46)	
Other	14.01(0.01)	12.45(1.02)	19.83(2.33)	
Education,%(SE)				0.03
High school or equivalent	24.20(0.02)	23.82(1.42)	25.59(2.39)	
Less than high school	9.84(0.01)	10.72(0.89)	6.56(1.11)	
Some college or above	65.97(0.03)	65.46(1.65)	67.85(2.57)	
Smoke,%(SE)				0.06
Former	30.65(0.02)	29.82(1.46)	33.75(3.34)	
Never	53.33(0.03)	55.03(1.94)	46.99(3.21)	
Now	16.02(0.01)	15.15(1.11)	19.26(2.18)	
Alcohol,%(SE)				0.19
Former	17.94(0.01)	17.89(1.11)	18.13(2.34)	
Heavy	15.56(0.01)	16.62(1.16)	11.61(1.96)	
Mild	42.89(0.03)	42.18(1.75)	45.55(2.60)	
Moderate	16.42(0.01)	15.88(0.92)	18.46(2.61)	
Never	7.19(0.01)	7.44(0.83)	6.25(1.48)	
Hypertension,%(SE)				< 0.001
No	52.95(0.03)	56.21(1.75)	40.78(2.90)	
Yes	47.05(0.02)	43.79(1.75)	59.22(2.90)	
Diabetes,%(SE)				0.38
Borderline	20.45(0.02)	20.94(1.21)	18.64(2.91)	
Yes	20.70(0.01)	19.88(1.04)	23.75(2.39)	
No	58.85(0.03)	59.18(1.38)	57.61(3.42)	
Physical activity level,%(SE)				0.33
High	39.79(0.02)	39.85(1.45)	39.55(2.68)	
Low	18.90(0.01)	18.18(1.10)	21.59(2.26)	
Middle	41.31(0.02)	41.97(1.49)	38.86(2.87)	
CVD,%(SE)				< 0.001
No	89.09(0.04)	90.98(0.89)	82.02(2.39)	
Yes	10.91(0.01)	9.02(0.89)	17.98(2.39)	
UIMQ,%(SE)				< 0.0001
Q1	20.74(0.01)	24.61(1.22)	6.30(1.25)	
Q2	23.14(0.02)	24.31(1.51)	18.78(2.26)	
Q3	26.98(0.02)	26.33(1.52)	29.41(2.59)	
Q4	29.13(0.02)	24.75(1.49)	45.51(2.63)	

OA, Osteoarthritis; UIM, Uric acid-Inflammation-Metabolic; UA, Uric Acid; BMI, Body Mass Index; WC, Waist circumference; eGFR, Estimated Glomerular Filtration Rate; HbA1c, Glycosylated hemoglobin; WBC, White blood cells; FPG, Fasting Plasma Glucose; HB, Hemoglobin; TG, Triglycerides; TC, Total cholesterol; HDL, High density lipoprotein; hs-CRP, High-sensitivity C-Reactive Protein; CVD, Cardiovascular disorders.

### 3.2 LASSO variable selection

**Figure 2:**
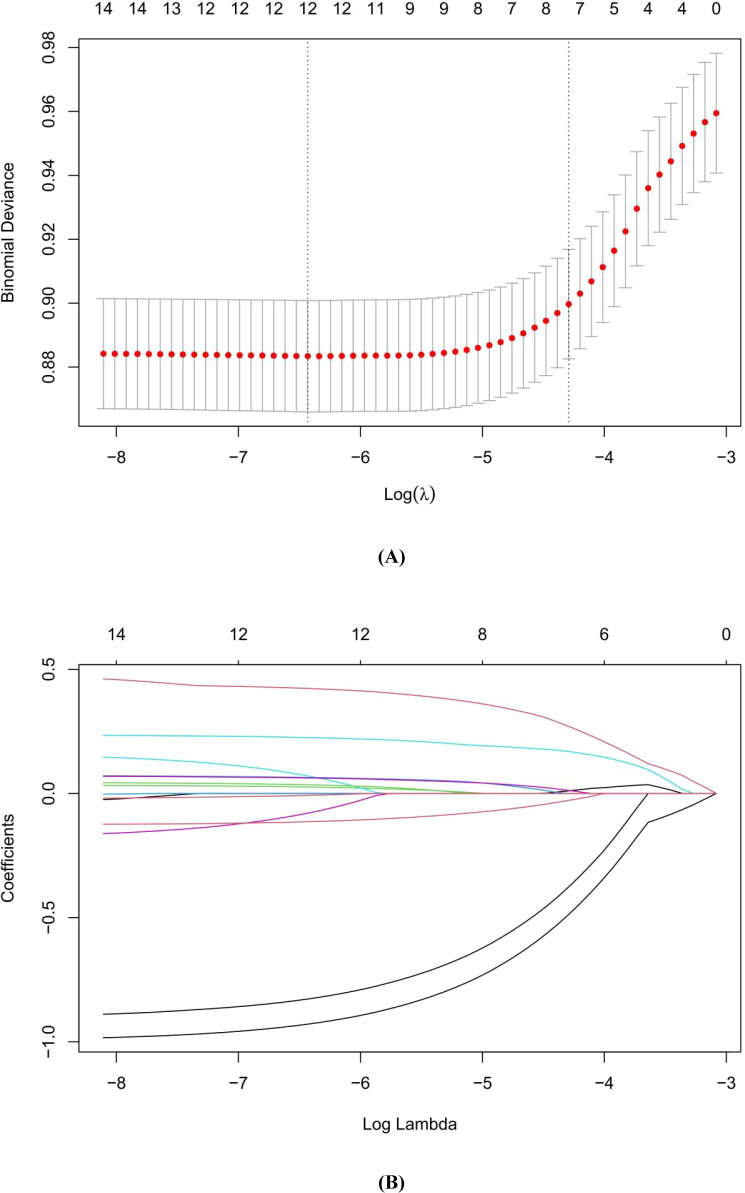
LASSO regression for variable selection. **(A)** Coefficient profile plot showing the shrinkage of 14 candidate variable coefficients as the regularization parameter λ increases. **(B)** Ten-fold cross-validation plot for optimal λ selection. The left dashed line represents the λ value with minimum cross-validated deviance (lambda.min), and the right dashed line represents the λ value within one standard error of the minimum (lambda.1se). Six variables with non-zero coefficients at lambda.1se were selected for the UIM score:UA,eGFR,hs-CRP,BMI,WC,Creatinine and HDL.

Cross-validation results from LASSO logistic regression revealed that at lambda.1se (λ = 0.014), 7 out of 14 candidate variables had non-zero coefficients and were selected for the UIM score model (**Figure 2A**). The seven selected variables and their LASSO coefficients were: uric acid (UA, β = -0.028), eGFR (β = -0.487), hs-CRP (β = 0.013), BMI (β = 0.011), waist circumference (WC, β = 0.270), creatinine (β = -0.376), and HDL-C (β = 0.169). The seven excluded variables (coefficients = zero) were: WBC count, hemoglobin, serum albumin, fasting plasma glucose, HbA1c, triglycerides, and total cholesterol. The LASSO coefficient path plot (**Figure 2B**) demonstrated that as the penalty parameter λ decreased, eGFR entered the model first (largest |β|), followed by creatinine, WC, HDL-C, hs-CRP, UA, and BMI, reflecting the relative importance of each variable for OA prediction.

### 3.3 UIM score construction

Based on the seven LASSO-selected variables, weighted multivariable logistic regression results are presented in **Table 2**. All seven variables maintained statistical significance in the fully adjusted model (all P < 0.05). The UIM score calculation formula was: UIM Score = -1.6643 - 0.1100×Z_UA - 1.0001×Z_eGFR + 0.0742×Z_hs-CRP + 0.4868×Z_WC + 0.2368×Z_HDL - 0.0518×Z_BMI - 0.9201×Z_Creatinine. The regression coefficients, OR values, and 95% CIs for each variable were as follows: each 1-SD increase in BMI was associated with a 5.0% increase in OA risk (OR = 1.05, 95% CI: 1.010-1.102); each 1-SD increase in hs-CRP increased OA risk by 7.7% (OR = 1.077, 95% CI: 1.012-1.175); each 1-SD increase in UA decreased OA risk by 10.4% (OR = 0.896, 95% CI: 0.798-0.995); each 1-SD increase in WC increased OA risk by 62.7% (OR = 1.627, 95% CI: 1.320-2.009); each 1-SD increase in HDL-C increased OA risk by 26.7% (OR = 1.267, 95% CI: 1.150-1.395); each 1-SD increase in eGFR decreased OA risk by 60.2% (OR = 0.398, 95% CI: 0.326-0.484); and each 1-SD increase in creatinine decreased OA risk by 63.2% (OR = 0.368, 95% CI: 0.312-0.432). The UIM score ranged from -10.092 to 1.405 in the study population. Participants were categorized into quartiles: Q1 (UIM Score ≤ -2.209), Q2 (-2.209 < UIM Score ≤ -1.664), Q3 (-1.664 < UIM Score ≤ -1.133), and Q4 (UIM Score > -1.133).

**Table 2:** Screening variables by LASSO regression and their parameter estimation in multivariate logistic regression.

Variables	LASSO coefficient (λ = 0.0137)	Regression coefficient β	OR	95%CI	P-value	VIF
UA	-0.016	-0.028	0.896	0.798-0.995	<0.05	2.31
eGFR	-0.127	-0.487	0.398	0.326-0.484	<0.05	1.42
Hs-CRP	0.003	0.013	1.077	1.012-1.175	<0.05	1.86
BMI	0.080	0.011	1.05	1.010-1.102	<0.05	1.28
WC	0.0.167	0.270	1.627	1.320-2.009	<0.05	2.14
Creatinine	-0.178	-0.376	0.368	-.312-0.432	<0.05	1.93
HDL	0.123	0.169	1.267	1.150-1.395	<0.05	2.08
Excluded variables (LASSO Coefficient = 0)						
WBC	0	–	–	–	–	–
HB	0	–	–	–	–	–
Albumin	0	–	–	–	–	–
FPG	0	–	–	–	–	–
HbA1c	0	–	–	–	–	–
TG	0	–	–	–	–	–
TC	0	–	–	–	–	–

All continuous variables were normalized to Z-score (using NHANES population mean and standard deviation) before LASSO and logistic regression, and hs-CRP was normalized after natural log transformation. Regression coefficients β and ORs are based on standardized variables and represent effect sizes per 1-SD increase. VIF: Variance inflation factor (VIF < 5 suggests no severe multicollinearity). Model adjusted variables: age, sex, race, education, smoking, alcohol consumption, physical activity, diabetes, hypertension, history of cardiovascular disease.

### 3.4 Association between UIM score and OA risk

#### 3.4.1 Primary logistic regression analysis

**Table 3** presents the associations between UIM score quartiles and OA prevalence risk. In the unadjusted model (Model 1), the Q4 group had a 7.18-fold higher OA risk compared to Q1 (OR = 7.18, 95% CI: 4.64-11.13, P < 0.001). Although the association attenuated with progressive adjustment for confounding factors, it remained highly significant: Model 2 (adjusted for age and sex): Q4 vs Q1 OR = 2.50 (95% CI: 1.39-4.49); Model 3 (further adjusted for lifestyle factors): Q4 vs Q1 OR = 2.71 (95% CI: 1.51-4.87); Model 4 (fully adjusted): Q4 vs Q1 OR = 2.63 (95% CI: 1.47-4.72, P < 0.001). In the fully adjusted model, Q2 and Q3 groups also showed significantly higher OA risk compared to Q1 (Q2: OR = 1.97, 95% CI: 1.12-3.46; Q3: OR = 2.34, 95% CI: 1.30-4.21), demonstrating a clear dose-response relationship (P for trend = 0.006). When UIM score was treated as a continuous variable in the fully adjusted model, each 1-SD increase was associated with an 84% higher OA prevalence risk (OR = 1.84, 95% CI: 1.36-2.48, P < 0.001).

**Table 3:** Multivariate logistic regression analysis of UIM score quartile group and risk of OA.

UIM score grouping	Model 1(unadjusted)OR (95% CI)	Model 2 (+ aage and gender) OR (95% CI)	Model 3 (+ lifestyle) OR (95% CI)	Model 4 (fully adjusted) OR (95% CI)
Continuous variable (/1-SD)	2.55(2.15,3.01)***	1.77(1.36-2.30)***	1.89(1.41-2.53)***	1.84(1.36-2.48)***
Q1(Reference)	Reference	Reference	Reference	Reference
Q2	3.02(1.74-5.25)***	2.04(1.13-3.69)**	2.02(1.14-3.55)**	1.97(1.12-3.46)**
Q3	4.36(2.68-7.11)***	2.31(1.29-4.13)**	2.40(1.33,4.33)**	2.34(1.30-4.21)**
Q4	7.18(4.64-11.13)***	2.50(1.39-4.49)**	2.71(1.51-4.87)**	2.63(1.47-4.72)**
P for trend	<0.0001	0.007	0.004	0.006

**P<0.01; ***P < 0.001. Model 1: not adjusted for any covariates; Model 2: adjusted for age (continuous variable) and sex; Model 3: adjusted for education, smoking status, drinking status, and physical activity level on the basis of Model 2; Model 4 (fully adjusted): further adjusted for diabetes, hypertension, and history of cardiovascular disease on the basis of Model 3. OR, odds ratio; CI, confidence interval; SD, standard deviation.

#### 3.4.2 Nonlinear dose-response relationship

**Figure 3:**
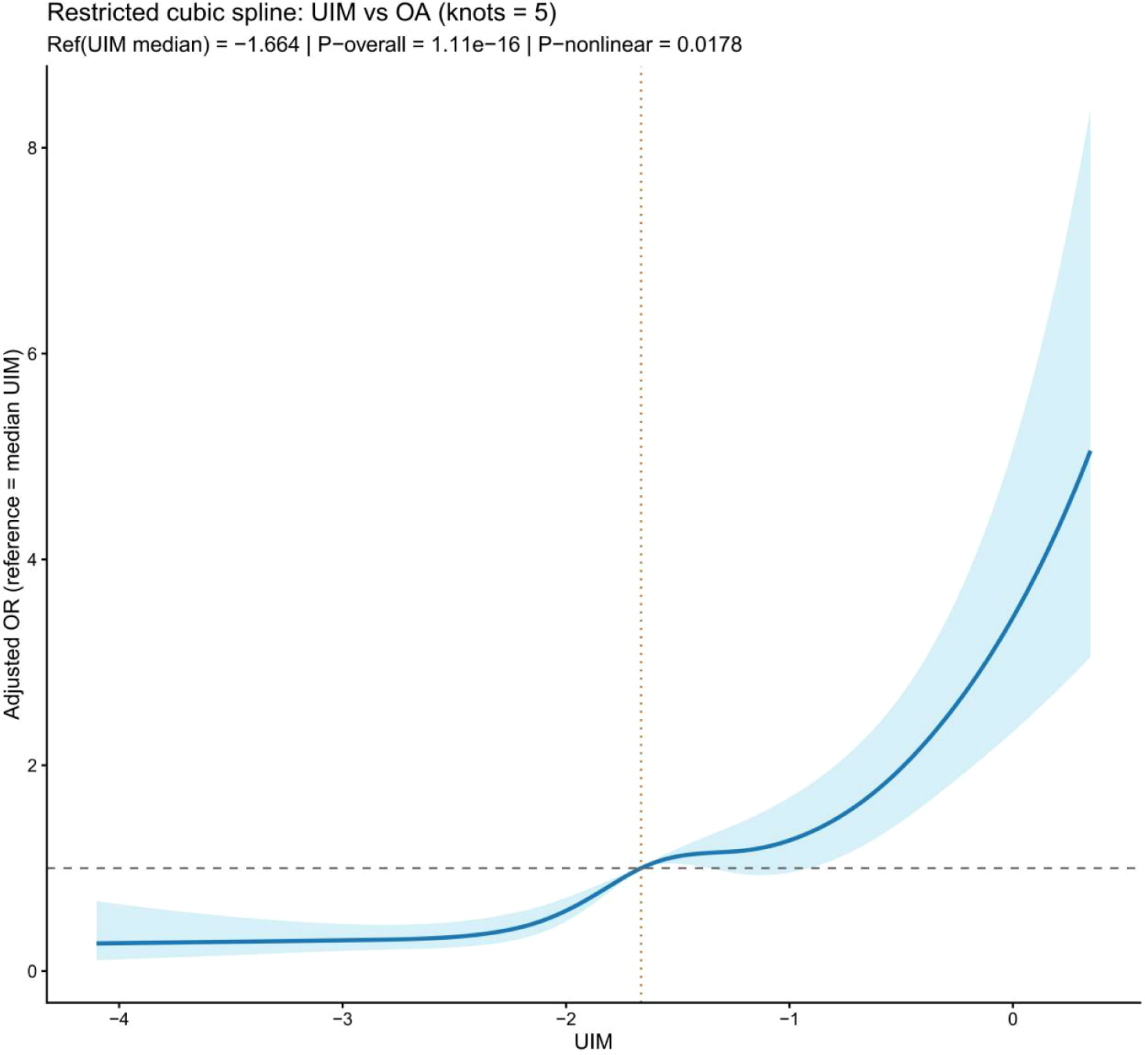
Restricted cubic spline (RCS) curve showing the non-linear dose-response relationship between UIM score and OA risk. Adjusted for age, sex, education, smoking, alcohol, physical activity, diabetes, hypertension, and CVD history. Knots were placed at the 5th, 35th, 65th, and 95th percentiles. The reference point was set at the median UIM score (0.05). The solid line represents the adjusted OR, and the shaded area represents the 95% CI. Overall association P<0.001; P for non-linearity = 0.0178.

RCS analysis (**Figure 3**) revealed a significant nonlinear relationship between UIM score and OA risk (P-overall < 0.001, P-nonlinear = 0.0178). The dose-response curve exhibited a “J-shaped” pattern: as UIM levels increased, the adjusted OR values showed a nonlinear upward trend. When UIM was below the reference value, OR values approached 1, suggesting no significant association between lower UIM levels and OA prevalence risk. However, when UIM exceeded the reference value, OR values increased significantly with rising UIM levels, with accelerating increments, indicating a close association between high-level UIM exposure and increased OA prevalence risk, with OR values reaching above 8. This threshold effect suggests that OA risk escalates sharply once multidimensional uric acid-inflammation-metabolism disturbances exceed a certain threshold.

#### 3.4.3 Subgroup analysis and interaction effects

**Figure 4:**
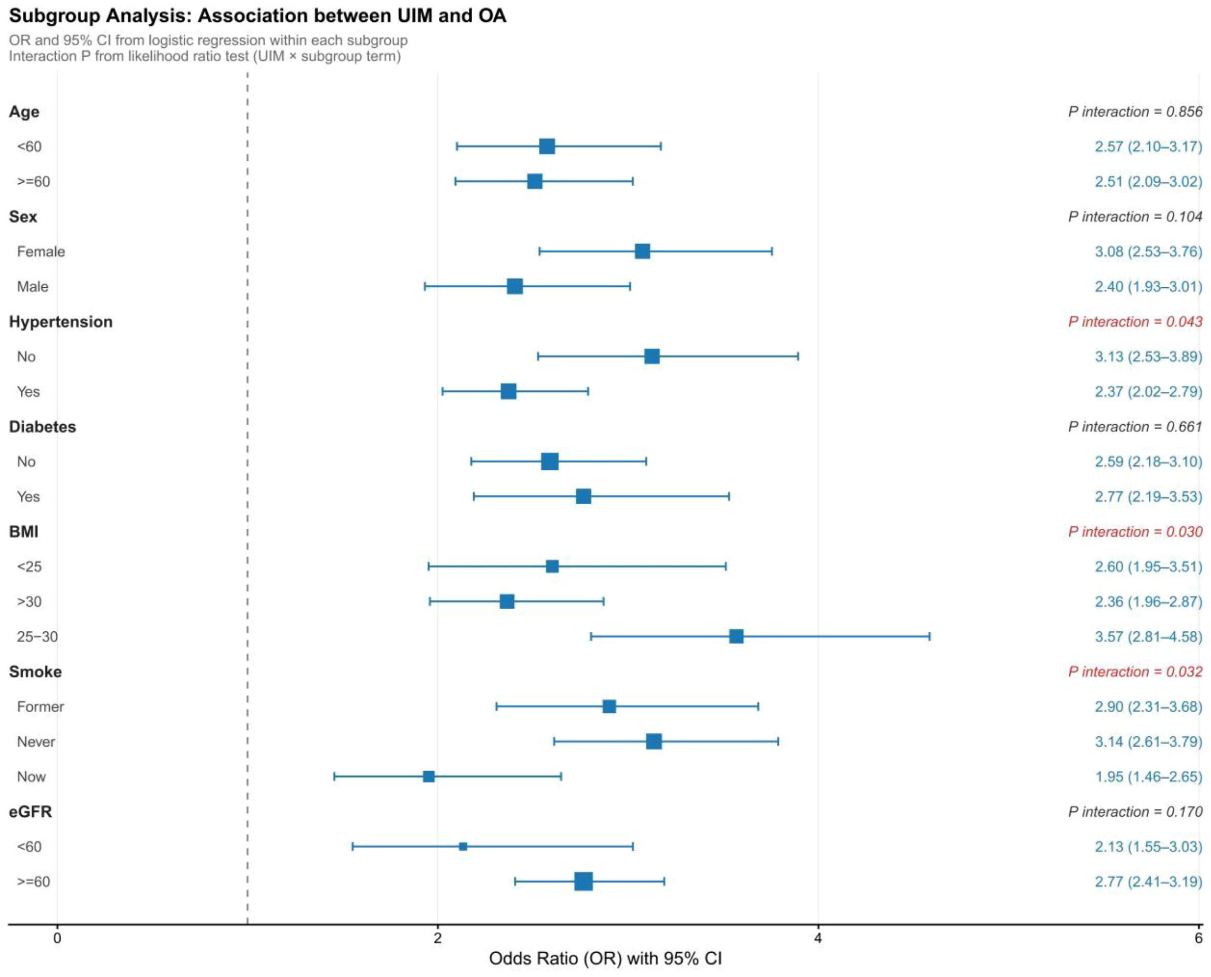
Forest plot showing the association between UIM score (per 1-SD increase) and OA risk across pre-specified subgroups (fully adjusted Model 4). OR and 95% CI are displayed for each subgroup. P for interaction was calculated by adding a multiplicative interaction term between UIM score and each subgroup variable.

Subgroup analysis results are presented as forest plots (**Figure 4**). Across all predefined subgroups, UIM score showed significant positive associations with OA risk, though effect sizes varied. Gender differences: The association between UIM score and OA was more pronounced in females (female: OR = 3.08, 95% CI: 2.53-3.76; male: OR = 2.40, 95% CI: 1.93-3.01; interaction P = 0.104). This finding may be related to exacerbated metabolic dysfunction and enhanced inflammatory responses following postmenopausal estrogen decline. BMI stratification: Among overweight individuals (BMI = 25-30 kg/m²), the impact of UIM score on OA risk was most prominent (OR = 3.57, 95% CI: 2.81-4.58), while the association was relatively weaker in the normal weight group (BMI < 25 kg/m²; OR = 2.60, 95% CI: 1.95-3.51; interaction P = 0.030), suggesting synergistic effects between metabolic dysfunction and obesity on OA risk. Age stratification: No significant difference in effect size was observed between < 60 years and ≥ 60 years groups (interaction P = 0.856), indicating stable predictive value of UIM score across different age groups. Diabetes stratification: An enhanced association trend between UIM score and OA was observed in diabetic patients (OR = 2.77 vs 2.59), though the interaction P value did not reach statistical significance (interaction P = 0.661). eGFR stratification: The effect size in the eGFR < 60 mL/min/1.73 m² group was lower than in the eGFR ≥ 60 group (OR = 2.13 vs 2.77), with interaction P = 0.170, not reaching significance. Significant interaction effects were observed in other subgroups (hypertension, smoking status) (interaction P < 0.05).

### 3.5 Predictive performance of UIM score

#### 3.5.1 Discrimination

**Figure 5:**
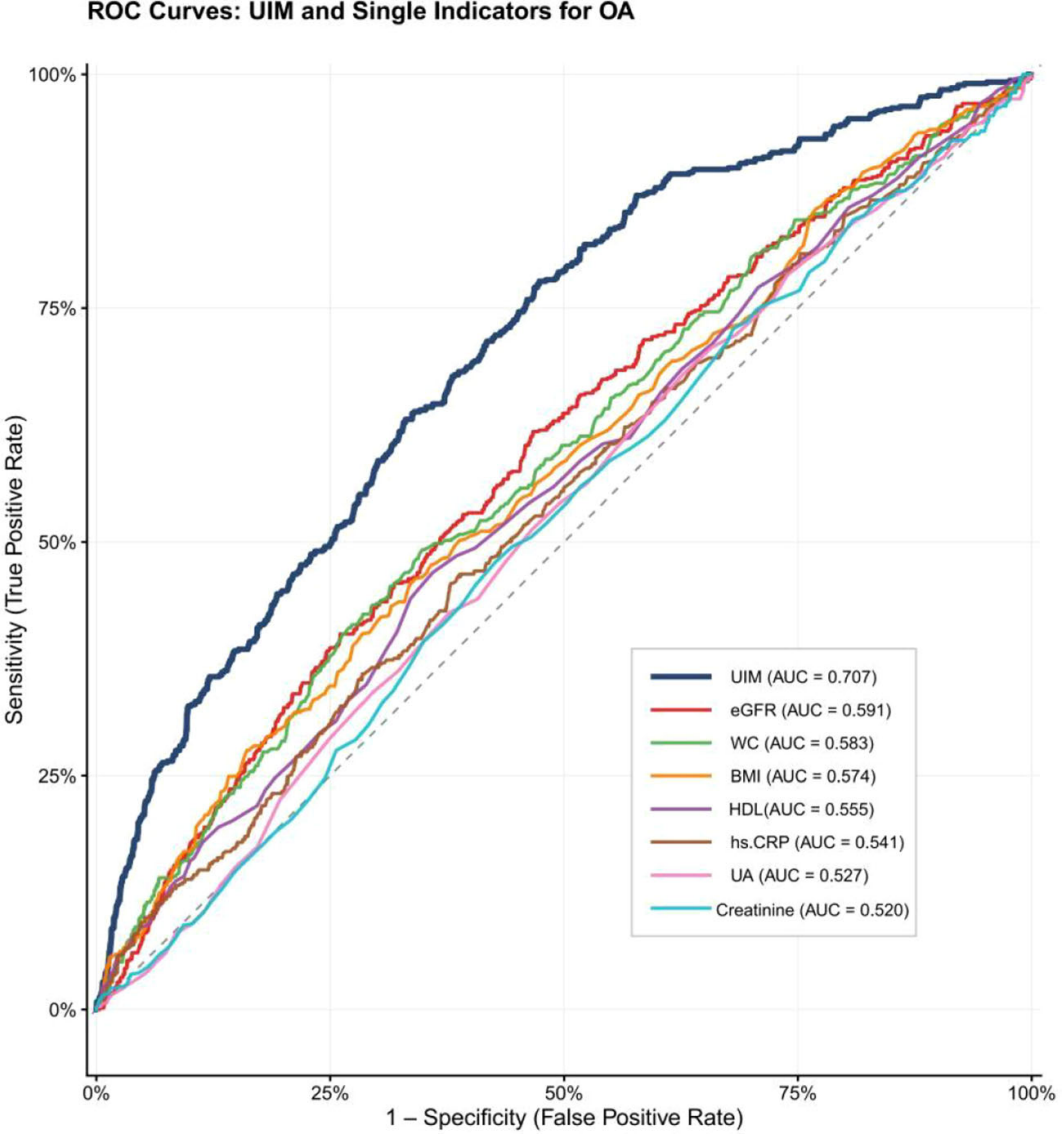
compares the discriminative abilities of the UIM score and individual biomarkers in predicting osteoarthritis, with the results presented in the form of receiver operating characteristic curves.

In the NHANES discovery cohort, the UIM score achieved an AUC of 0.707 (95% CI: 0.684-0.729) for OA prediction, significantly outperforming all individual indicators: UA had an AUC of only 0.527 (95% CI: 0.502-0.552), hs-CRP 0.541 (95% CI: 0.516-0.567), BMI 0.574 (95% CI: 0.548-0.599), eGFR 0.591 (95% CI: 0.566-0.616), WC 0.583 (95% CI: 0.557-0.608), creatinine 0.520 (95% CI: 0.492-0.545), and HDL-C 0.555 (95% CI: 0.529-0.580). DeLong tests demonstrated statistically significant differences between the UIM score AUC and each individual indicator (all P < 0.001) (**Figure 5**; **Table 4**). Compared to the baseline model containing only traditional risk factors (age + sex + BMI) with an AUC of 0.694, incorporating the UIM score significantly improved the AUC to 0.727 (DeLong P = 0.037). The NRI was 0.183 (95% CI: 0.131-0.235, P = 0.039) and IDI was 0.047 (95% CI: 0.039-0.055, P = 0.043), indicating incremental association value of the UIM score.

**Table 4:** Comparison of predictive efficacy of UIM score and each single index for OA.

Predictors	AUC	95%CI	Sensitivity(%)	Specificity(%)	Youden index	ΔAUC compared to UIM score	DeLong P-value	NRI	IDI
UIM Score	0.707	0.684-0.729	71.8	65.4	0.372	–	–	–	–
UA	0.527	0.502-0.552	63.2	50.8	0.140	-0.180	<0.001	–	–
eGFR	0.591	0.566-0.616	59.3	57.4	0.167	-0.116	<0.001	–	–
Hs-CRP	0.541	0.516-0.567	60.4	58.3	0.187	-0.166	<0.001	–	–
BMI	0.574	0.548-0.599	65.8	58.6	0.244	-0.133	<0.001	–	–
WC	0.583	0.557-0.608	54.7	56.8	0.201	-0.124	<0.001	–	–
Creatinine	0.520	0.492-0.545	57.6	56.2	0.138	-0.187	<0.001	–	–
HDL	0.555	0.529-0.580	58.1	60.2	0.147	-0.152	<0.001	–	–
Traditional Risk Model①	0.694	0.625-0.763	68.4	62.1	0.305	-0.013	<0.001	–	–
Traditional model + UIM score	0.727	0.534-0.985	73.2	67.8	0.410	+0.020②	0.037②	0.183**	0.047**

① The traditional risk model contained three variables: age, gender and BMI; ② ΔAUC and DeLong P values compared with the traditional risk model. DeLong ‘s method was used for AUC comparisons. NRI (net weight classification improvement index) and IDI (comprehensive discriminant improvement index) were incremental association values of UIM score compared with the traditional risk model,**P < 0.05. Optimal cut points were determined based on Youden index (sensitivity + specificity − 1 maximization).

#### 3.5.2 Calibration

**Figure 6:**
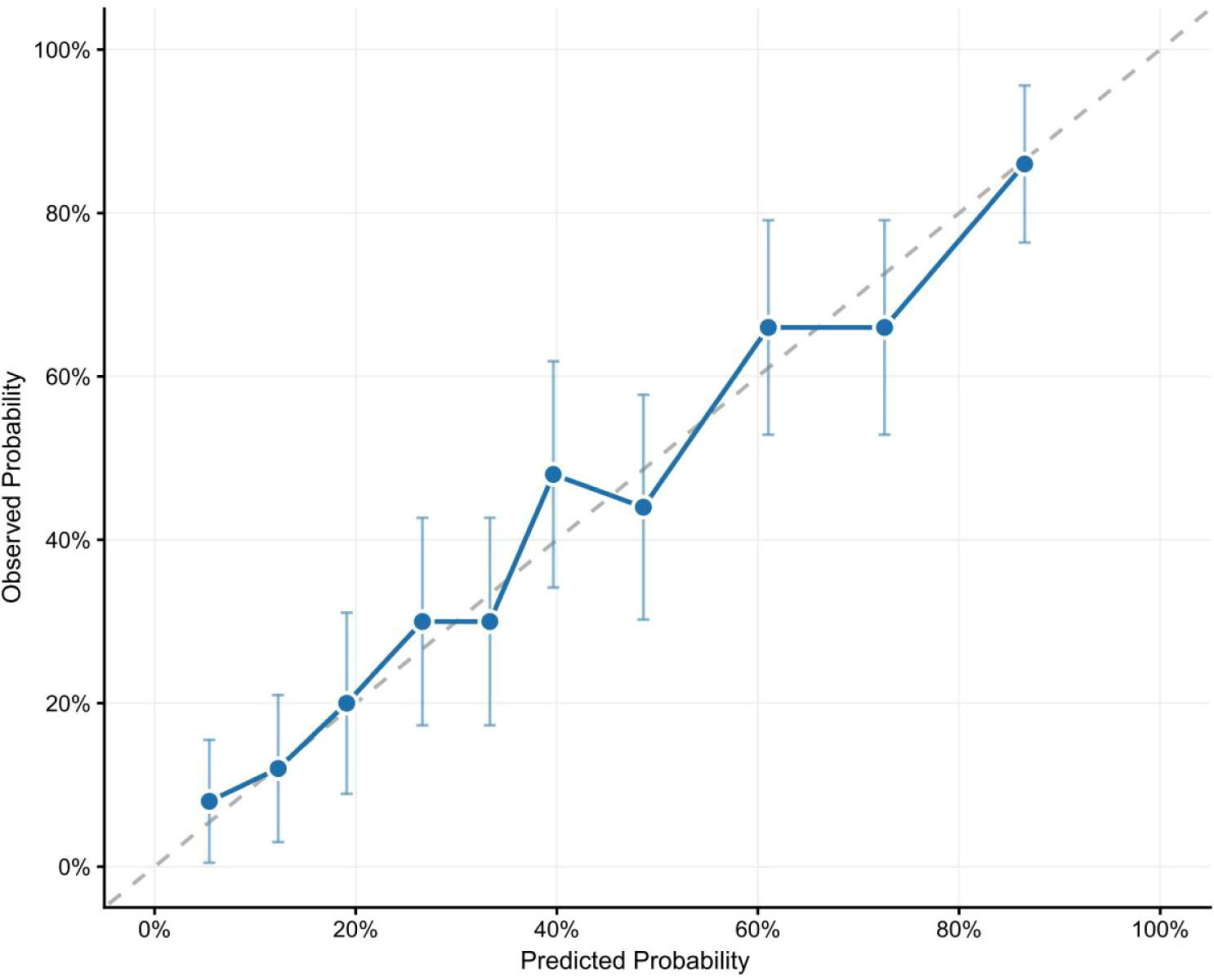
Calibration plots of the UIM score model. The x-axis represents the predicted probability, and the y-axis represents the observed probability. The diagonal dashed line represents perfect calibration.

The Hosmer-Lemeshow test demonstrated good calibration of the UIM score model (χ² = 9.82, P = 0.278), with no significant deviation between predicted and observed probabilities. The calibration curve (**Figure 6**) showed excellent agreement between predicted and actual probabilities, with a calibration slope of 0.96 (ideal value = 1), calibration intercept of 0.008 (ideal value = 0), and mean absolute error (MAE) of 0.023.

#### 3.5.3 Decision curve analysis

**Figure 7:**
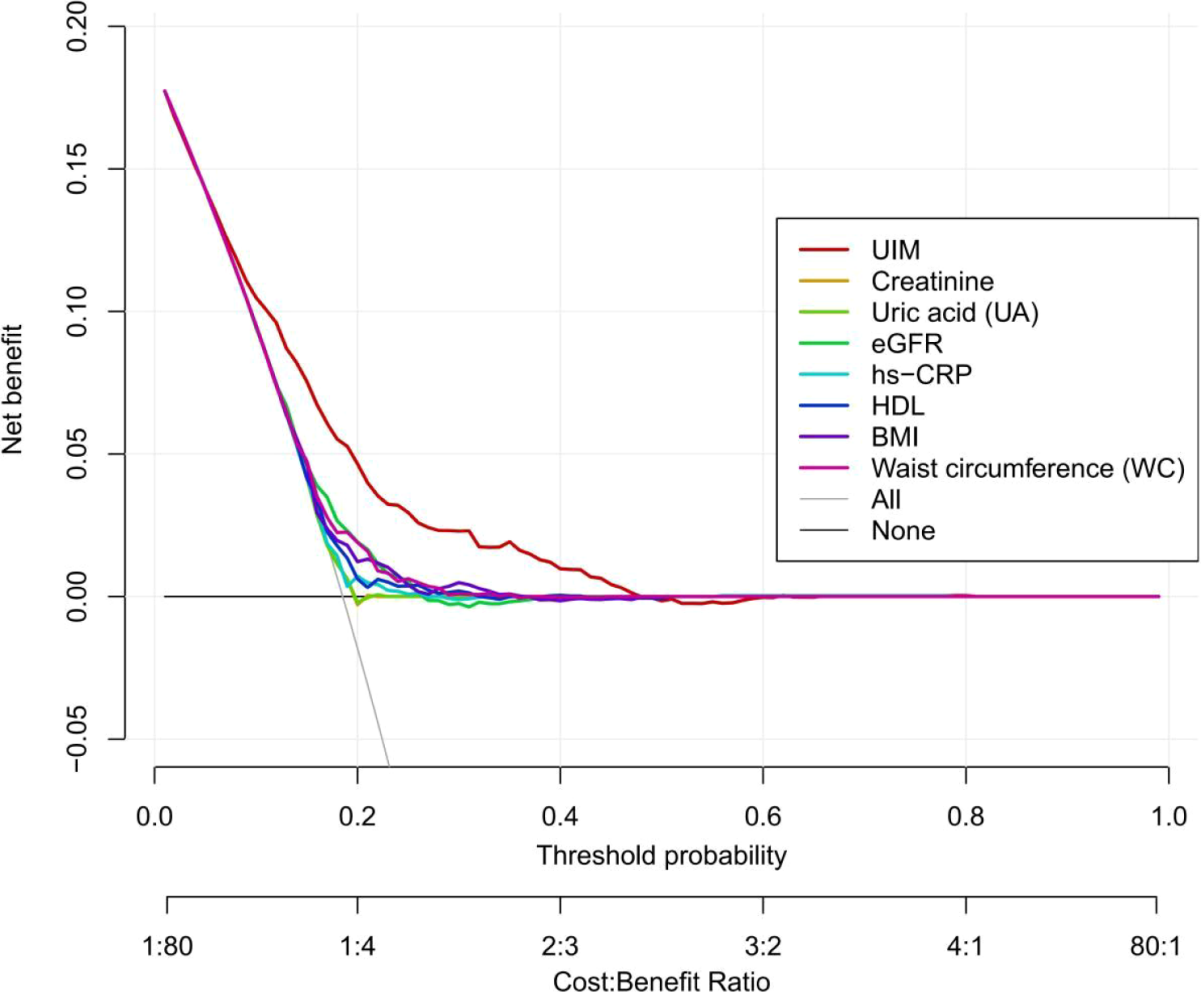
Decision curve analysis (DCA) comparing the net clinical benefit of the UIM score model versus individual biomarker models and two extreme strategies (“treat all” and “treat none”). The x-axis represents the threshold probability, and the y-axis represents the net benefit.

DCA results (**Figure 7**) demonstrated that across threshold probabilities of 5%-45%, the UIM score model consistently provided higher net benefit than both “treat all” and “treat none” strategies, and consistently outperformed any individual indicator model. Particularly within the clinically most relevant threshold probability range of 15%-40%, the net benefit advantage of the UIM score was most pronounced, indicating good practical value for clinical OA risk screening.

### 3.6 Three-dimensional contribution analysis

**Figure 8:**
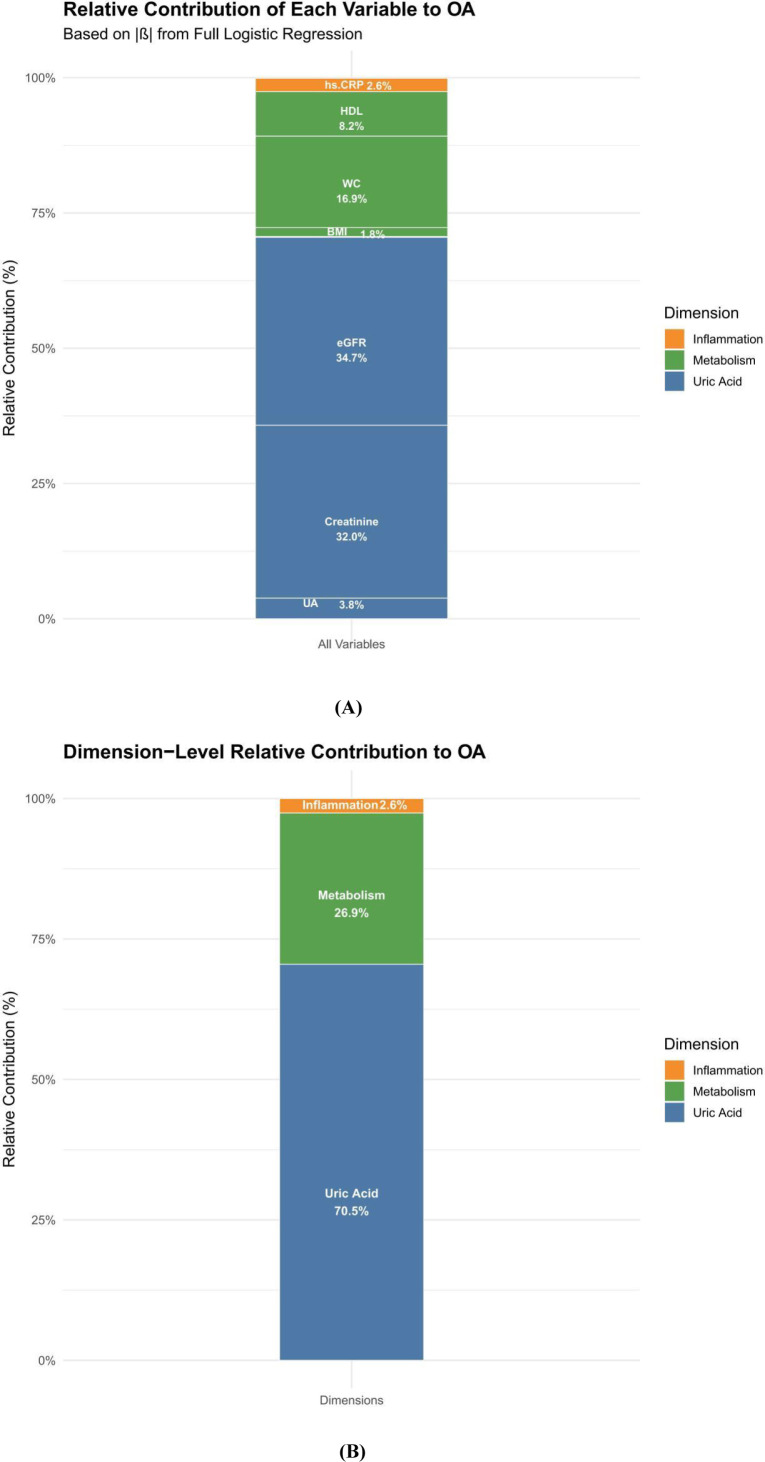
The contribution of uric acid, inflammation, and metabolic factors to the ability of the UIM score to predict osteoarthritis. **(A)** Stacked bar chart showing the relative contribution of each variable to OA. **(B)** Stacked bar chart showing the relative weight of each factor (i.e., their proportion of the total β coefficient sum).

**Figure 8**; **Table 5** present the relative contributions of the uric acid, inflammatory, and metabolic dimensions to the UIM score’s predictive performance. The uric acid dimension contributed most significantly, accounting for 70.50% of total predictive performance (sub-score OR = 2.68, 95% CI: 2.30-3.13, P < 0.001), with the integration of creatinine and eGFR reflecting a more comprehensive uric acid metabolic status. The metabolic dimension contributed 26.92% (sub-score OR = 2.71, 95% CI: 2.21-3.32, P < 0.001), with WC making a particularly prominent contribution. The inflammatory dimension accounted for 2.58% (sub-score OR = 5.36, 95% CI: 1.92-14.96, P < 0.001).

**Table 5:** Independent association of each dimension subscore with OA risk (fully adjusted model, NHANES).

Dimensions	Subscore OR (/1-SD) (95% CI)	P-value	Relative contribution%
Uric acid dimension	2.68(2.30-3.13)	<0.001	70.50
Inflammation dimension	5.36(1.92-14.96)	<0.001	2.58
Metabolic dimension	2.71(2.21-3.32)	<0.001	26.92
UIM score	1.84(1.36-2.48)	<0.001	100

**Figure 9:**
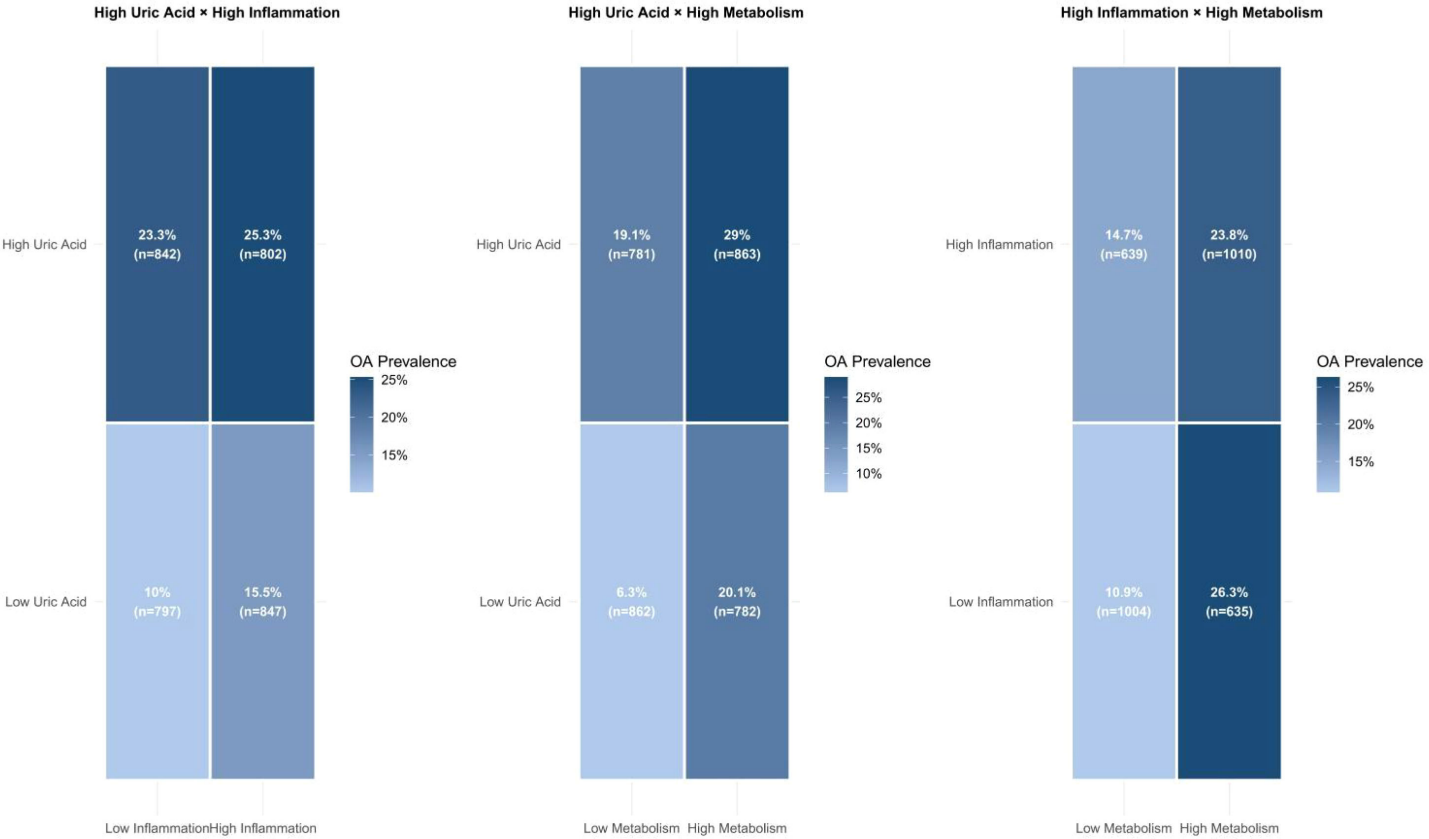
The interactive effects between uric acid, inflammation, and metabolic disorders in pairs.

To evaluate pairwise interaction effects among the three dimensions of uric acid, inflammation, and metabolic dysfunction, this study employed relative excess risk due to interaction (RERI), attributable proportion (AP), and synergy index (SI) to quantitatively analyze additive scale interactions, while using multiplicative interaction term P-values to assess multiplicative scale interaction effects. Stratified prevalence results (**Figure 9**; **Table 6**) showed that in the high uric acid × high inflammation combination, the OA prevalence rates for low uric acid/low inflammation, low uric acid/high inflammation, high uric acid/low inflammation, and high uric acid/high inflammation groups were 10.0% (n = 797), 15.5% (n = 847), 23.3% (n = 842), and 25.3% (n = 802), respectively, demonstrating a clear increasing trend with rising uric acid and inflammation levels. In the high uric acid × high metabolic dysfunction combination, the four groups’ OA prevalence rates were 6.3% (n = 862), 20.1% (n = 782), 19.1% (n = 781), and 29.0% (n = 863), respectively, with the highest OA prevalence in the combined high uric acid and high metabolic dysfunction exposure group. In the high inflammation × high metabolic dysfunction combination, the four groups’ OA prevalence rates were 10.9% (n = 1004), 26.3% (n = 635), 14.7% (n = 639), and 23.8% (n = 1010), respectively. Notably, the high inflammation alone group had higher prevalence (26.3%) than the combined high inflammation and high metabolic dysfunction group (23.8%), preliminarily suggesting potential antagonistic effects between the two.

**Table 6:** Interdimensional superadditive interaction effect.

Interaction dimension combination	RERI	95%CI	P-value	AP	SI
Hyperinflammation×Hypermetabolic disorder	-0.787	-1.601--0.086	0.0113	-0.307	0.665
Hyperuricemia×hyperinflammation	-0.322	-1.142-0.428	0.0435	-0.106	0.864
Hyperuricemia×Hypermetabolic disorder	-0.184	-1.819-1.089	0.0001	-0.030	0.965

The definition of “high” for each dimension was based on the median of the corresponding subscore (ie, subscore > median defined as”high”). RERI (relative excess risk) = OR_11_−OR_10_−OR_01_ + 1, and RERI > 0 indicates a superadditive interaction, i.e., a combined effect greater than the sum of two independent effects.Attribution Ratio (AP),Interaction Index (SI).

Quantitative interaction analysis further confirmed these findings. On the multiplicative scale, all three dimensional combinations showed statistically significant interaction effects (high uric acid × high inflammation: P = 0.0435; high uric acid × high metabolic dysfunction: P = 0.0001; high inflammation × high metabolic dysfunction: P = 0.0113). On the additive scale, high uric acid and high inflammation showed RERI = -0.322 (95% CI: -1.142 to 0.428), AP = -0.106, SI = 0.864, with 95% CI spanning 0, indicating no statistically significant additive interaction; high uric acid and high metabolic dysfunction showed RERI = -0.184 (95% CI: -1.819 to 1.089), AP = -0.030, SI = 0.965, with 95% CI also spanning 0, indicating no statistically significant additive interaction; high inflammation and high metabolic dysfunction showed RERI = -0.787 (95% CI: -1.601 to -0.086), AP = -0.307, SI = 0.665, with 95% CI not spanning 0, indicating statistically significant additive antagonistic effect (P = 0.0113). This suggests that the combined effect of both exposures on OA prevalence risk is significantly lower than the sum of their individual independent effects, with approximately 30.7% of OA cases from combined exposure attributable to the “offsetting” effect of this antagonistic interaction. In summary, the combined effects among the three dimensions are predominantly antagonistic, with the additive antagonistic effect between high inflammation and high metabolic dysfunction being most prominent, suggesting that under conditions of high metabolic dysfunction, the additional contribution of inflammation to OA incidence risk may be somewhat attenuated, indicating inherent complexity in the joint pathogenic mechanisms of the three dimensions for OA prevalence risk.

### 3.7 External validation

Using the coefficients and standardization parameters from NHANES modeling, UIM scores were calculated for each participant in the validation cohort. Results showed that the highest quartile (Q4) of UIM score had a 2.21-fold higher osteoarthritis risk compared to Q1 (fully adjusted OR = 2.21, 95% CI: 1.74-2.73, P = 0.03), with direction and magnitude highly consistent with the NHANES discovery cohort findings. Each 1-SD increase in UIM score yielded an OR of 1.92 (95% CI: 1.11-3.30, P = 0.02), with trend test P = 0.376. ORs for Q2 and Q3 groups were 1.99 (95% CI: 0.40-9.96) and 2.23 (95% CI: 1.81-2.73), respectively (**Table 7**).

**Table 7:** Association of UIM score with arthritis risk: external validation results for validation cohort.

UIM score grouping	Model 1(unadjusted)OR (95% CI)	Model 2 (+ age and gender) OR (95% CI)	Model 3 (+ lifestyle) OR (95% CI)	Model 4 (fully adjusted) OR (95% CI)
Continuous variable (/1-SD)	2.92(2.10-4.05)***	1.83(1.08-3.10)**	1.96(1.65-3.89)**	1.92(1.11-3.30)**
Q1(Reference)	Reference	Reference	Reference	Reference
Q2	4.15(0.84-20.45)*	2.53(0.43-4.75)*	2.19(1.42-2.83)**	1.99(0.40-9.96)*
Q3	6.27(1.61-24.48)**	2.59(1.45-4.78)**	2.37(.44-3.60)**	2.23(1.81-2.73)**
Q4	10.72(3.24-35.45)***	2.49(1.38-6.10)***	2.37(1.39-4.28)**	2.21(1.74-2.73)**
P for trend	<0.0001	0.367	0.435	0.376

*P>0.05, **P<0.05, ***P < 0.001. Model 1: not adjusted for any covariates; Model 2: adjusted for age (continuous variable) and sex; Model 3: adjusted for education, smoking status, drinking status, and physical activity level on the basis of Model 2; Model 4 (fully adjusted): further adjusted for diabetes, hypertension, and history of cardiovascular disease on the basis of Model 3. OR: odds ratio; CI: confidence interval; SD: standard deviation.

### 3.8 Sensitivity analyses

A series of sensitivity analyses (**Table 8**) confirmed the robustness of the study findings (1): After excluding participants with hs-CRP > 10 mg/L (n = 273), Q4 vs Q1 OR = 2.52 (95% CI: 1.25-5.10), consistent with the primary analysis results (2). After excluding participants with BMI < 18.5 kg/m² (n = 24), Q4 vs Q1 OR = 2.59 (95% CI: 2.17-3.09), demonstrating stable results (3). After substituting the CKD-EPI 2021 creatinine formula for eGFR calculation, the association between UIM score and OA remained virtually unchanged (Q4 vs Q1 OR = 2.61, 95% CI: 2.19-3.11) (4). After IPTW analysis, Q4 vs Q1 OR = 2.47 (95% CI: 2.02-3.02), further excluding residual confounding effects (5). NHANES internal validation (70% training set/30% test set): training set Q4 vs Q1 OR = 2.68 (95% CI: 2.21-3.25), internal test set Q4 vs Q1 OR = 2.54 (95% CI: 1.96-3.29), with minimal differences, indicating no model overfitting.

**Table 8:** Summary of sensitivity analysis results (NHANES finding cohort, Q4 vs Q1 fully adjusted OR).

Sensitivity analysis	Sample size	OR(95%CI)	P-value
Main analysis (reference)	3288	2.63(1.47-4.72)	<0.001
① Exclude hs-CRP > 10 mg/L	3015	2.52(1.25-5.10)	0.010
② Exclude BMI < 18.5 kg/m ²	3264	2.59(2.17-3.09)	0.002
③Replace CKD-EPI 2021 formula to calculate eGFR	3288	2.61(2.19-3.11)	<0.001
④IPTW weighted analysis	3288	2.47(2.02-3.02)	<0.001
⑤Internal validation (70% training set)	2302	2.68(2.21-3.25)	<0.001
⑥Internal validation (30% test set)	986	2.54(1.96-3.29)	<0.001

IPTW (inverse probability treatment weighting) calculates stabilization weights based on propensity scores (estimated based on age, sex, race, education, smoking, alcohol consumption, physical activity, diabetes, hypertension, and history of CVD) for participants in Q1 and Q4 groups. All sensitivity analyses used the same fully adjusted model as the main analysis (Model 4).

## 4 Discussion

The present study constructed and validated a novel multidimensional composite score—the Uric acid-Inflammation-Metabolism (UIM) score—based on three biological dimensions encompassing uric acid metabolism, inflammatory status, and metabolic disturbance, and systematically evaluated its association with osteoarthritis (OA) prevalence. The principal findings are as follows (1): the UIM score demonstrated an independent, significant positive association with OA prevalence, exhibiting a “J-shaped” nonlinear dose-response relationship (2); the discriminative ability of the UIM score (AUC = 0.707) significantly surpassed that of all individual biomarkers and traditional risk factor models (3); this association remained robust across the external validation cohort and multiple sensitivity analyses; and (4) the predominant antagonistic interactions among the three dimensions revealed the inherent complexity of multidimensional OA pathogenic mechanisms. These findings provide a novel multidimensional biomarker reference for epidemiological assessment of OA prevalence and population-based risk grouping. Further high-level clinical evidence is still required to confirm its formal clinical application value and practical intervention guiding significance.

The pathogenesis of OA is multifactorial and involves multiple pathways, making single biomarkers insufficient to comprehensively capture its complex pathophysiological processes (26). Previous studies have independently reported associations between aberrant uric acid metabolism (27), chronic low-grade inflammation, metabolic syndrome (28), and OA prevalence. However, attempts to integrate these dimensions into a composite score are exceedingly rare. The present study used LASSO regression to select seven optimal predictors from 14 candidate variables, including UA, eGFR, serum creatinine, hs-CRP, waist circumference (WC), HDL-C, and BMI. We then constructed the UIM score using weighted multivariable logistic regression. The LASSO method shrinks variable coefficients through the L1 penalty term. It effectively mitigates multicollinearity while achieving model parsimony, and has been widely applied in variable selection for clinical assessment models (29). Notably, eGFR and serum creatinine entered the model first in the LASSO coefficient path plot and had the largest absolute regression coefficients. This suggests that the uric acid metabolism dimension—particularly renal filtration function plays a central role in OA prevalence assessment. This finding is consistent with previously reported associations between renal function impairment and articular cartilage degeneration (30).

In the association analysis between UIM score and OA prevalence, the fully adjusted model showed that participants in the highest quartile (Q4) of UIM score had a 2.63-fold higher OA prevalence than those in the lowest quartile (Q1) (OR = 2.63, 95% CI: 1.47–4.72). A significant dose-response relationship was also observed (P for trend = 0.006). This result aligns with prior research on the association between metabolic syndrome scores and OA (31). RCS analysis further revealed a “J-shaped” nonlinear relationship between UIM score and OA prevalence. Once the UIM score exceeded a certain threshold, OA prevalence increased sharply with rising UIM levels, with OR values exceeding 8 at the highest levels. This threshold phenomenon reflects the cumulative effect of multidimensional metabolic-inflammatory-uric acid disturbances in OA pathological progression, which is consistent with the “multiple-hit” hypothesis of disease causation (32). Similar nonlinear dose-response patterns have been reported in obesity-related OA (33) and metabolic syndrome-associated OA (34), further supporting the biological plausibility of our findings. At present, this threshold can only serve as a preliminary epidemiological reference index, and cannot be directly regarded as a definite clinical intervention cut-off value.

Baseline characteristics analysis showed that the OA group had significantly higher mean age, female proportion, BMI, waist circumference, total cholesterol, HDL-C, and prevalence of hypertension and CVD compared to non-OA controls. In contrast, eGFR, serum creatinine, and uric acid levels were lower in the OA group. Extensive epidemiological evidence has well established the associations between OA and advanced age, female sex, and obesity (1, 35). Of particular interest, the OA group in our study had paradoxically lower uric acid levels than the non-OA group. This seemingly counterintuitive phenomenon may be attributed to three factors: First, eGFR was significantly lower in the OA group. This suggests that renal impairment-induced reduction in uric acid excretion may paradoxically reflect decreased uric acid production or altered distribution in the context of compromised renal function. Second, as an endogenous antioxidant, uric acid may exert protective effects on cartilage under certain conditions. Its reduction may attenuate the oxidative stress defense of articular cartilage (10). Third, the inherent limitations of cross-sectional study designs preclude definitive inference on the direction of association, which is further discussed below.

Notably, our study found a positive association between HDL-C and OA prevalence, which contradicts some existing literature. This unexpected finding may be explained by HDL-C functional heterogeneity: in the context of metabolic disturbance (common in our OA cohort with high BMI and metabolic syndrome), HDL-C can become dysfunctional, losing its anti-inflammatory and cartilage-protective properties and instead promoting synovial inflammation and chondrocyte damage, thereby increasing OA risk. Further prospective studies are needed to clarify the causal relationship and verify this mechanism.

Regarding assessment performance, the UIM score achieved an AUC of 0.707 (95% CI: 0.684–0.729), indicating only moderate discriminative ability in distinguishing between individuals with and without OA. While this AUC is significantly higher than that of all seven individual biomarkers (all P < 0.001), such statistical superiority cannot be directly converted into obvious dominant value in actual clinical decision-making. Compared to the baseline model containing only traditional risk factors (AUC = 0.694), incorporating the UIM score improved the AUC to 0.727, with an NRI of 0.183 and IDI of 0.047. Although the improvement reached statistical significance, the overall promotion range was limited, and the actual clinical application benefit remained relatively limited. These findings are highly consistent with prior studies demonstrating the superiority of multidimensional composite scores over single indicators (36, 37). Notably, although the AUC of UA alone was merely 0.527, integrating the full spectrum of uric acid metabolism indicators (UA + eGFR + serum creatinine) substantially enhanced the assessment performance of the uric acid dimension sub-score (OR = 2.68), further corroborating the superiority of multidimensional integration strategies over single-indicator analyses. The model demonstrated good calibration (Hosmer-Lemeshow P = 0.278, calibration slope = 0.96, MAE = 0.023), and DCA showed that the UIM score provided certain net clinical benefit within partial threshold intervals. Nevertheless, based on its moderate predictive efficacy, the UIM score is not qualified to be used as an independent core tool for large-scale population OA screening in current clinical practice (38). It can only be used as one auxiliary reference index.

Subgroup analysis results demonstrated that the positive association between UIM score and OA prevalence was maintained consistently across all predefined subgroups, though effect sizes varied. In BMI stratification, the association was most prominent among overweight individuals (BMI = 25–30 kg/m²; OR = 3.57), with a significant interaction effect compared to the normal weight group (interaction P = 0.030), suggesting synergistic associations between metabolic dysfunction and overweight on OA prevalence. The dual mechanisms through which obesity is associated with OA progression—increased mechanical joint loading and promotion of systemic low-grade inflammation—are well supported by pathophysiological evidence (39). The association between UIM score and OA tended to be stronger in the female subgroup (OR = 3.08 vs. male OR = 2.40); although the interaction P value did not reach statistical significance (P = 0.104), this trend is consistent with the biological mechanisms of exacerbated metabolic dysfunction, enhanced inflammatory responses, and diminished cartilage-protective effects resulting from postmenopausal estrogen decline (40). The UIM score showed no significant difference in effect size between age groups (<60 years vs. ≥60 years; interaction P = 0.856), suggesting relatively stable association trend across age strata. It can provide auxiliary reference for OA related epidemiological analysis in middle-aged and elderly populations, rather than serving as targeted grouping basis for clinical intervention.

In view of the limited predictive performance and cross-sectional research nature of this study, we refrain from putting forward definite and standardized clinical prescriptive intervention suggestions based solely on UIM score values. The J-shaped association trend observed in this study can only enrich the theoretical understanding of OA multidimensional pathogenesis, and cannot be directly used to formulate unified diagnosis, treatment and health management plans for clinical patients. Relevant lifestyle adjustment and disease intervention measures still need to be formulated comprehensively combined with clinical symptoms, imaging manifestations and professional doctors’ clinical experience, rather than relying on this scoring result alone. The clinical net benefit reflected by DCA only confirms that this score has certain auxiliary reference value in partial crowd assessment scenarios, but cannot prove that it can effectively optimize clinical screening strategies and significantly improve early diagnosis efficiency in actual medical work.

Three-dimensional contribution analysis revealed the internal dimensional structure of the UIM score. The uric acid dimension contributed the most (70.50%), followed by the metabolic dimension (26.92%), while the inflammatory dimension contributed relatively modestly (2.58%). The dominant role of the uric acid dimension is consistent with prior reports that highlight the importance of renal function indices in OA assessment (30). It also reflects the ability of integrated eGFR and serum creatinine to comprehensively capture uric acid metabolic status. Although the inflammatory dimension contributed the least, its sub-score had the highest OR value (OR = 5.36). This indicates that the independent predictive ability of inflammatory markers (hs-CRP) at elevated levels for OA prevalence should not be overlooked. This aligns with pathological mechanism research on OA synovitis, where chronic low-grade inflammation plays an important role in OA cartilage degeneration. It does so by activating chondrocyte apoptosis and the expression of matrix-degrading enzymes (41).

Analysis of interdimensional interaction effects provided important pathophysiological insights. A statistically significant additive antagonistic effect was found between high inflammation and high metabolic disturbance (RERI = −0.787, AP = −0.307, SI = 0.665, P = 0.011). This means the combined association of both exposures on OA prevalence is significantly lower than the sum of their individual independent associations. Approximately 30.7% of OA cases attributed to combined exposure are “offset” by this antagonistic interaction. This finding contradicts simple synergistic pathogenesis hypotheses. It suggests that the additional association of inflammation with OA prevalence may be somewhat weakened in the context of high metabolic disturbance. One possible explanation is that in patients with metabolic syndrome, adipose tissue secretes compensatory anti-inflammatory adipokines (such as adiponectin). These may partially counteract the direct damaging effects of inflammatory pathways on articular cartilage (42). Alternatively, metabolic dysfunction itself is independently associated with OA progression. It does this by increasing mechanical loading and reducing adequate cartilage nutritional supply, thereby diminishing the relative proportional association of inflammatory signaling. This finding indicates that multidimensional OA pathogenic mechanisms are not simple linear superpositions. Instead, they exhibit inherent complexity and interdimensional regulatory effects, which have important scientific implications for understanding the pathogenesis of heterogeneous OA subtypes.

External validation results further demonstrated the relatively stable association trend of the UIM score across different crowds. In the Anhui Provincial Hospital validation cohort (n = 859), the Q4 group of UIM score had a 2.21-fold higher osteoarthritis prevalence than the Q1 group (OR = 2.21, 95% CI: 1.74–2.73, P = 0.03). The direction and magnitude were highly consistent with the NHANES discovery cohort. Such consistent association trends preliminarily reflect that the correlation between uric acid metabolism, inflammation, metabolic disturbance and OA has certain population universality. A series of sensitivity analyses further confirmed the robustness of the study conclusions. Excluding participants with acute inflammation (hs-CRP > 10 mg/L) and underweight (BMI < 18.5 kg/m²), substituting the eGFR calculation formula, IPTW propensity score weighting, and internal validation (ΔAUC = 0.011) did not substantially alter the primary study conclusions. This further rules out the possibility of result bias and model overfitting.

Several other limitations of this study warrant attention. First, the cross-sectional design of NHANES precludes causal inference, and prospective studies are needed to further verify the actual predictive effect of the UIM score on incident OA. Second, there is a significant inconsistency in OA diagnostic criteria between the two cohorts, which introduces potential misclassification bias and limits their direct comparability this discrepancy is primarily due to the inherent characteristics of the data sources. OA diagnosis in NHANES was based on self-reported questionnaires without radiographic confirmation or specialist evaluation, while the validation cohort adopted strict ACR diagnostic standards confirmed by specialists. This difference may affect the stability of research results and restrict the unified popularization of this scoring system. Third, although the present study adjusted for multiple potential confounders (including demographic variables, lifestyle factors, and comorbidities), as an observational study it cannot entirely exclude unmeasured confounding variables that may affect the observed associations. Dietary factors, related medication use, OA severity status and hormonal status were not fully included in the adjustment range, which may bring residual confounding bias to the research conclusions. Fourth, the finding that HDL-C levels were higher in the OA group than in the non-OA group differs from some existing literature, and its biological mechanisms require further investigation. Fifth, the trend test P value in the validation cohort (P = 0.376) did not reach statistical significance, potentially attributable to the relatively limited sample size, differences in OA diagnostic criteria between cohorts and differences in disease severity between the two populations. Sixth, the inflammatory dimension of the UIM score has obvious limitations: it only contains a single index, the correlation effect value is unstable, and the independent prediction value is insufficient, which suggests that this dimension needs to be further optimized and supplemented in subsequent research. Finally, the moderate discriminative ability of the UIM score and the lack of direct comparison with existing mature OA prediction tools mean that its practical promotion space in clinical daily work is still limited, and any excessive optimistic evaluation of its application value should be avoided.

In conclusion, this study constructed and validated the UIM composite score integrating three dimensions of uric acid metabolism, inflammatory status, and metabolic disturbance. This score demonstrates an independent, significant, and nonlinear association with OA prevalence, with assessment performance surpassing all individual biomarkers, and exhibits good robustness of association trend across different population subgroups and cohorts. The predominantly antagonistic interaction patterns among the three dimensions reveal the inherent complexity of multidimensional OA pathogenic mechanisms. Restricted by cross-sectional research design, moderate model discrimination efficiency and many unresolved limitations, the UIM score can only be positioned as a novel auxiliary epidemiological evaluation indicator for OA prevalence status and population risk grouping at this stage, and cannot be used as mature clinical prediction tool and early screening tool for OA onset. This study mainly enriches the theoretical research basis of OA multidimensional pathogenesis. More standardized, large-sample, multicenter prospective researches are still needed in the future to continuously optimize this scoring system and verify its actual clinical application value, so as to provide more reliable evidence support for OA precision prevention and auxiliary assessment.

## Data Availability

The original contributions presented in the study are included in the article/supplementary material. Further inquiries can be directed to the corresponding author.

## References

[1] Hunter DJ Bierma-Zeinstra S . Osteoarthritis. Lancet (London England). (2019) 393:1745–59. doi: 10.1016/j.berh.2011.11.008 31034380

[2] GBD 2019 Diseases and Injuries Collaborators. Global burden of 369 diseases and injuries in 204 countries and territories, 1990-2019: a systematic analysis for the Global Burden of Disease Study 2019. Lancet (London England). (2020) 396:1204–22. doi: 10.1016/s0140-6736(20)30925-9 33069326 PMC7567026

[3] Safiri S Kolahi AA Smith E Hill C Bettampadi D Mansournia MA . Global, regional and national burden of osteoarthritis 1990-2017: a systematic analysis of the Global Burden of Disease Study 2017. Ann Rheumatic Dis. (2020) 79:819–28. doi: 10.1136/annrheumdis-2019-216515 32398285

[4] Loeser RF Goldring SR Scanzello CR Goldring MB . Osteoarthritis: a disease of the joint as an organ. Arthritis Rheumatism. (2012) 64:1697–707. doi: 10.1002/art.34453 22392533 PMC3366018

[5] Zhuo Q Yang W Chen J Wang Y . Metabolic syndrome meets osteoarthritis. Nat Rev Rheumatol. (2012) 8:729–37. doi: 10.1038/nrrheum.2012.135 22907293

[6] Courties A Sellam J Berenbaum F . Metabolic syndrome-associated osteoarthritis. Curr Opin Rheumatol. (2017) 29:214–22. doi: 10.1097/bor.0000000000000373 28072592

[7] Berenbaum F . Osteoarthritis as an inflammatory disease (osteoarthritis is not osteoarthrosis)! Osteoarthritis Cartilage. (2013) 21:16–21. doi: 10.1016/j.joca.2012.02.621 23194896

[8] Sokolove J Lepus CM . Role of inflammation in the pathogenesis of osteoarthritis: latest findings and interpretations. Ther Adv Musculoskeletal Dis. (2013) 5:77–94. doi: 10.1177/1759720x12467868 23641259 PMC3638313

[9] Martinon F Pétrilli V Mayor A Tardivel A Tschopp J . Gout-associated uric acid crystals activate the NALP3 inflammasome. Nature. (2006) 440:237–41. doi: 10.1038/nature04516 16407889

[10] Sautin YY Johnson RJ . Uric acid: the oxidant-antioxidant paradox. Nucleosides Nucleotides Nucleic Acids. (2008) 27:608–19. doi: 10.1080/15257770802138558 18600514 PMC2895915

[11] Monira Hussain S Wang Y Cicuttini FM Simpson JA Giles GG Graves S . Incidence of total knee and hip replacement for osteoarthritis in relation to the metabolic syndrome and its components: a prospective cohort study. Semin Arthritis Rheumatism. (2014) 43:429–36. doi: 10.1016/j.semarthrit.2013.07.013 24012045

[12] Niu J Clancy M Aliabadi P Vasan R Felson DT . Metabolic syndrome, its components, and knee osteoarthritis: the framingham osteoarthritis study. Arthritis Rheumatol (Hoboken NJ). (2017) 69:1194–203. doi: 10.1002/art.40087 28257604 PMC5449217

[13] Dalbeth N Merriman TR Stamp LK . Gout. Lancet (London England). (2016) 388:2039–52. doi: 10.1093/med/9780199642489.003.0141 27112094

[14] Boer CG Hatzikotoulas K Southam L Stefánsdóttir L Zhang Y Coutinho de Almeida R . Deciphering osteoarthritis genetics across 826,690 individuals from 9 populations. Cell. (2021) 184:4784–4818.e17. doi: 10.1016/j.cell.2021.11.003 34450027 PMC8459317

[15] Bouillanne O Morineau G Dupont C Coulombel I Vincent JP Nicolis I . Geriatric Nutritional Risk Index: a new index for evaluating at-risk elderly medical patients. Am J Clin Nutr. (2005) 82:777–83. doi: 10.1093/ajcn/82.4.777 16210706

[16] de Munter W Blom AB Helsen MM Walgreen B van der Kraan PM Joosten LA . Cholesterol accumulation caused by low density lipoprotein receptor deficiency or a cholesterol-rich diet results in ectopic bone formation during experimental osteoarthritis. Arthritis Res Ther. (2013) 15:R178. doi: 10.1186/ar4367 24286458 PMC3978425

[17] Clockaerts S Bastiaansen-Jenniskens YM Runhaar J Van Osch GJ Van Offel JF Verhaar JA . The infrapatellar fat pad should be considered as an active osteoarthritic joint tissue: a narrative review. Osteoarthritis Cartilage. (2010) 18:876–82. doi: 10.1016/j.joca.2010.03.014 20417297

[18] Johnson CL Paulose-Ram R Ogden CL Carroll MD Kruszon-Moran D Dohrmann SM . National health and nutrition examination survey: analytic guidelines, 1999-2010. Vital Health Stat Ser 2 Data Eval Methods Res. (2013) 161:1–24. 25090154

[19] Zhao Y Hu Y Smith JP Strauss J Yang G . Cohort profile: the China health and retirement longitudinal study (CHARLS). Int J Epidemiol. (2014) 43:61–8. doi: 10.1093/ije/dys203 23243115 PMC3937970

[20] Friedman J Hastie T Tibshirani R . Regularization paths for generalized linear models via coordinate descent. J Stat Software. (2010) 33:1–22. doi: 10.18637/jss.v033.i01 PMC2929880 20808728

[21] Desquilbet L Mariotti F . Dose-response analyses using restricted cubic spline functions in public health research. Stat Med. (2010) 29:1037–51. doi: 10.1002/sim.3841 20087875

[22] DeLong ER DeLong DM Clarke-Pearson DL . Comparing the areas under two or more correlated receiver operating characteristic curves: a nonparametric approach. Biometrics. (1988) 44:837–45. doi: 10.2307/2531595 3203132

[23] Vickers AJ Elkin EB . Decision curve analysis: a novel method for evaluating prediction models. Med Decision Making: Int J Soc For Med Decision Making. (2006) 26:565–74. doi: 10.1177/0272989x06295361 17099194 PMC2577036

[24] Pencina MJ D'Agostino RB Sr. Steyerberg EW . Extensions of net reclassification improvement calculations to measure usefulness of new biomarkers. Stat Med. (2011) 30:11–21. doi: 10.1002/sim.4085 21204120 PMC3341973

[25] Altman R Asch E Bloch D Bole G Borenstein D Brandt K . Development of criteria for the classification and reporting of osteoarthritis. Classification of osteoarthritis of the knee. Diagnostic and Therapeutic Criteria Committee of the American Rheumatism Association. Arthritis Rheumatism. (1986) 29:1039–49. doi: 10.3109/03009748709102175 3741515

[26] Mobasheri A Batt M . An update on the pathophysiology of osteoarthritis. Ann Phys Rehabil Med. (2016) 59:333–9. doi: 10.1016/j.rehab.2016.07.004 27546496

[27] Roddy E Doherty M . Gout and osteoarthritis: a pathogenetic link? Joint Bone Spine. (2012) 79:425–7. doi: 10.1016/j.jbspin.2012.03.013 22867976

[28] Courties A Gualillo O Berenbaum F Sellam J . Metabolic stress-induced joint inflammation and osteoarthritis. Osteoarthritis Cartilage. (2015) 23:1955–65. doi: 10.1016/j.joca.2015.05.016 26033164

[29] Steyerberg EW Harrell FE . Prediction models need appropriate internal, internal-external, and external validation. J Clin Epidemiol. (2016) 69:245–7. doi: 10.1016/j.jclinepi.2015.04.005 25981519 PMC5578404

[30] Veronese N Stubbs B Solmi M Smith TO Noale M Cooper C . Association between lower limb osteoarthritis and incidence of depressive symptoms: data from the osteoarthritis initiative. Age Ageing. (2017) 46:470–6. doi: 10.1093/ageing/afw216 27932358

[31] Zheng H Chen C . Body mass index and risk of knee osteoarthritis: systematic review and meta-analysis of prospective studies. BMJ Open. (2015) 5:e007568. doi: 10.1136/bmjopen-2014-007568 26656979 PMC4679914

[32] Felson DT Niu J Clancy M Sack B Aliabadi P Zhang Y . Effect of recreational physical activities on the development of knee osteoarthritis in older adults of different weights: the Framingham Study. Arthritis Rheumatism. (2007) 57:6–12. doi: 10.1002/art.22464 17266077

[33] Pottie P Presle N Terlain B Netter P Mainard D Berenbaum F . Obesity and osteoarthritis: more complex than predicted! Ann Rheumatic Dis. (2006) 65:1403–5. doi: 10.1136/ard.2006.061994 17038451 PMC1798356

[34] Louati K Vidal C Berenbaum F Sellam J . Association between diabetes mellitus and osteoarthritis: systematic literature review and meta-analysis. RMD Open. (2015) 1:e000077. doi: 10.1136/rmdopen-2015-000077 26535137 PMC4613158

[35] Srikanth VK Fryer JL Zhai G Winzenberg TM Hosmer D Jones G . A meta-analysis of sex differences prevalence, incidence and severity of osteoarthritis. Osteoarthritis Cartilage. (2005) 13:769–75. doi: 10.1016/j.joca.2005.04.014 15978850

[36] Collins GS Reitsma JB Altman DG Moons KG . Transparent reporting of a multivariable prediction model for individual prognosis or diagnosis (TRIPOD): the TRIPOD statement. BMJ (Clinical Res Ed). (2015) 350:g7594. doi: 10.1016/j.jclinepi.2014.11.010 25569120

[37] Alba AC Agoritsas T Walsh M Hanna S Iorio A Devereaux PJ . Discrimination and calibration of clinical prediction models: Users' guides to the medical literature. Jama. (2017) 318:1377–84. doi: 10.1001/jama.2017.12126 29049590

[38] Vickers AJ Cronin AM Elkin EB Gonen M . Extensions to decision curve analysis, a novel method for evaluating diagnostic tests, prediction models and molecular markers. BMC Med Inf Decis Making. (2008) 8:53. doi: 10.1186/1472-6947-8-53 19036144 PMC2611975

[39] Bliddal H Leeds AR Christensen R . Osteoarthritis, obesity and weight loss: evidence, hypotheses and horizons - a scoping review. Obes Reviews: Off J Int Assoc For Study Obes. (2014) 15:578–86. doi: 10.1111/obr.12173 24751192 PMC4238740

[40] Roman-Blas JA Castañeda S Largo R Herrero-Beaumont G . Osteoarthritis associated with estrogen deficiency. Arthritis Res Ther. (2009) 11:241. doi: 10.1186/ar2791 19804619 PMC2787275

[41] Knights AJ Redding SJ Maerz T . Inflammation in osteoarthritis: the latest progress and ongoing challenges. Curr Opin Rheumatol. (2023) 35:128–34. doi: 10.1097/bor.0000000000000923 36695054 PMC10821795

[42] Conde J Scotece M Gómez R López V Gómez-Reino JJ Lago F . Adipokines: biofactors from white adipose tissue. A complex hub among inflammation, metabolism, and immunity. BioFactors (Oxford England). (2011) 37:413–20. doi: 10.1002/biof.185 22038756

